# Dynamic changes of urinary microbiota in patients with bladder cancer after surgery and its clinical significance

**DOI:** 10.3389/fimmu.2025.1638628

**Published:** 2025-09-01

**Authors:** Ye Lang, Pei Li, Ruixiang He, Bo Zhu, Guang Wang, Jiongming Li

**Affiliations:** Department of Urology, The Second Affiliated Hospital, Kunming Medical University, Kunming, China

**Keywords:** bladder cancer, urinary microbiota, ileal neobladder reconstruction, dynamic changes, clinical significance

## Abstract

Bladder cancer is one of the most common malignancies of the urogenital system, with a high incidence and mortality. The treatment of bladder cancer is diverse, with surgical treatment being the most common approach, including transurethral resection of bladder tumor and radical cystectomy. Following radical cystectomy, patients often undergo ileal neobladder reconstruction to restore urinary storage and voiding functions. However, postoperative changes in the urinary microbiota have become a major issue for bladder cancer patients. Traditionally, urine was believed to be sterile, but an increasing body of research has demonstrated the presence of a resident microbiota in urine, which is closely associated with the development of bladder diseases, postoperative complications, and patient prognosis. Dynamic changes in the urinary microbiota may lead to urinary tract infections, tumor recurrence, and other issues, severely affecting patients’ recovery and quality of life. In recent years, with the advancement of high-throughput sequencing technology, research on the urinary microbiota has deepened, particularly regarding its changes and clinical significance after bladder cancer surgery. Although studies have explored the impact of urinary microbiota on recurrence and prognosis after bladder cancer surgery, research on urinary microbiota changes following ileal neobladder reconstruction is still limited. Therefore, this review aims to summarize the latest research on the dynamic changes of urinary microbiota in bladder cancer patients postoperatively, especially focusing on changes after ileal neobladder reconstruction, providing references for clinical treatment and future research directions.

## 1 Introduction

Bladder cancer is a common malignant tumor of the urinary system and is one of the top ten most prevalent cancers worldwide, with approximately 550,000 new cases diagnosed each year (1, 2). According to the Global Cancer Statistics 2020, there are approximately 573,278 new cases and 212,536 deaths from bladder cancer in 2020, with the incidence in men significantly higher than in women, placing a substantial burden on global public health systems (3). The latest GLOBOCAN data reveal that bladder cancer accounts for 3% of all cancer diagnoses globally, with a particularly high prevalence in developed countries (4). In recent years, with continuous advancements in diagnostic technologies, early screening and diagnosis of bladder cancer have gradually gained attention. However, the high recurrence rate and metastatic nature of bladder cancer continue to pose significant challenges in its treatment.

Studies have shown that the high incidence of bladder cancer is closely associated with smoking habits, industrial pollution, and occupational exposure to carcinogenic substances (5–7). Smoking is considered one of the most significant risk factors for bladder cancer. Evidence suggests that smokers have a substantially higher risk of developing bladder cancer compared to non-smokers (8). Carcinogens present in cigarette smoke, such as polycyclic aromatic hydrocarbons and aromatic amines, can damage the DNA of bladder epithelial cells, thereby promoting tumorigenesis (9). Despite the increasing implementation of tobacco control policies, the global prevalence of smoking remains high, which continues to be a major contributor to the burden of bladder cancer. In addition to smoking, environmental factors also play a critical role in the pathogenesis of bladder cancer (10). Certain occupational groups, such as workers in the chemical manufacturing, petrochemical, and dye industries, are at higher risk of exposure to aromatic amines, which have been strongly linked to the development of bladder cancer (11). Moreover, the incidence of bladder cancer increases with age. The majority of patients are over 60 years old, with those aged 70 and above being particularly susceptible (12). This age-dependent incidence may be attributed to age-related declines in immune function, reduced DNA repair capacity, and prolonged exposure to environmental carcinogens.

Currently, bladder cancer treatment options are heterogeneous, with surgery being one of the most important therapeutic approaches (13). The choice of surgical treatment depends on the stage and pathological characteristics of the bladder cancer. There are two main types of bladder cancer: non-muscle invasive bladder cancer and muscle-invasive bladder cancer (14). For non-muscle invasive bladder cancer, the tumor is confined to the bladder mucosa or submucosa without invading the muscular layer of the bladder wall. The standard surgical treatment for this stage is transurethral resection of bladder tumor, followed by risk stratification-based adjuvant intravesical therapy, with an overall survival rate of 90% (15). For muscle-invasive bladder cancer, where the tumor has invaded the bladder muscular layer, the standard treatment method is radical cystectomy combined with urinary diversion. However, the cure rate remains low due to various factors (15, 16). Ileal neobladder reconstruction is a common form of urinary diversion, where the ileum is used to replace the resected bladder, thus restoring the patient’s urinary storage and voiding functions (17). In contemporary clinical practice, ileal neobladder reconstruction has gained widespread adoption, establishing itself as a cornerstone technique for bladder cancer treatment across multidisciplinary medical institutions.

Postoperative changes in the urinary microbiota represent a significant issue for bladder cancer patients (18). Traditionally, urine was believed to be sterile; however, increasing evidence suggests that a resident microbiota exists in urine and influences the development of various bladder diseases, such as overactive bladder, interstitial cystitis, neurogenic bladder, and other bladder disorders (19). Recent studies have found that the urinary microbiota is closely associated with bladder cancer and may serve as potential biomarkers and therapeutic targets for the disease (20–22). Following cystectomy and urinary diversion, the structure and function of the urinary microbiota may undergo significant changes. These alterations may be closely related to the occurrence of postoperative complications, patients’ quality of life, and long-term prognosis (23). Research indicates that changes in the urinary microbiota may lead to a range of issues, including urinary tract infections and tumor recurrence, all of which severely affect patients’ recovery and quality of life (24). In recent years, with the development of high-throughput sequencing technology, research on the urinary microbiota has deepened (23). The dynamic changes in the urinary microbiota and its interactions with the host have become a research hotspot in the field of urological surgery. However, studies on the dynamic changes of the urinary microbiota and its clinical significance in bladder cancer patients postoperatively are still insufficient, particularly regarding the changes in the urinary microbiota after ileal neobladder reconstruction and its impact on patient prognosis, which requires further exploration.

Therefore, this review aims to summarize the research progress on the dynamic changes of urinary microbiota in bladder cancer patients postoperatively, with a particular focus on the changes in the urinary microbiota after ileal neobladder reconstruction and its clinical significance. This narrative review intends to provide a theoretical basis for clinical treatment and offer insights for future research directions.

## 2 Characteristics of the urinary microbiota in patients with bladder cancer before surgery

### 2.1 The burden of bladder cancer disease and regional differences in urine microbiota

**Figure 1:**
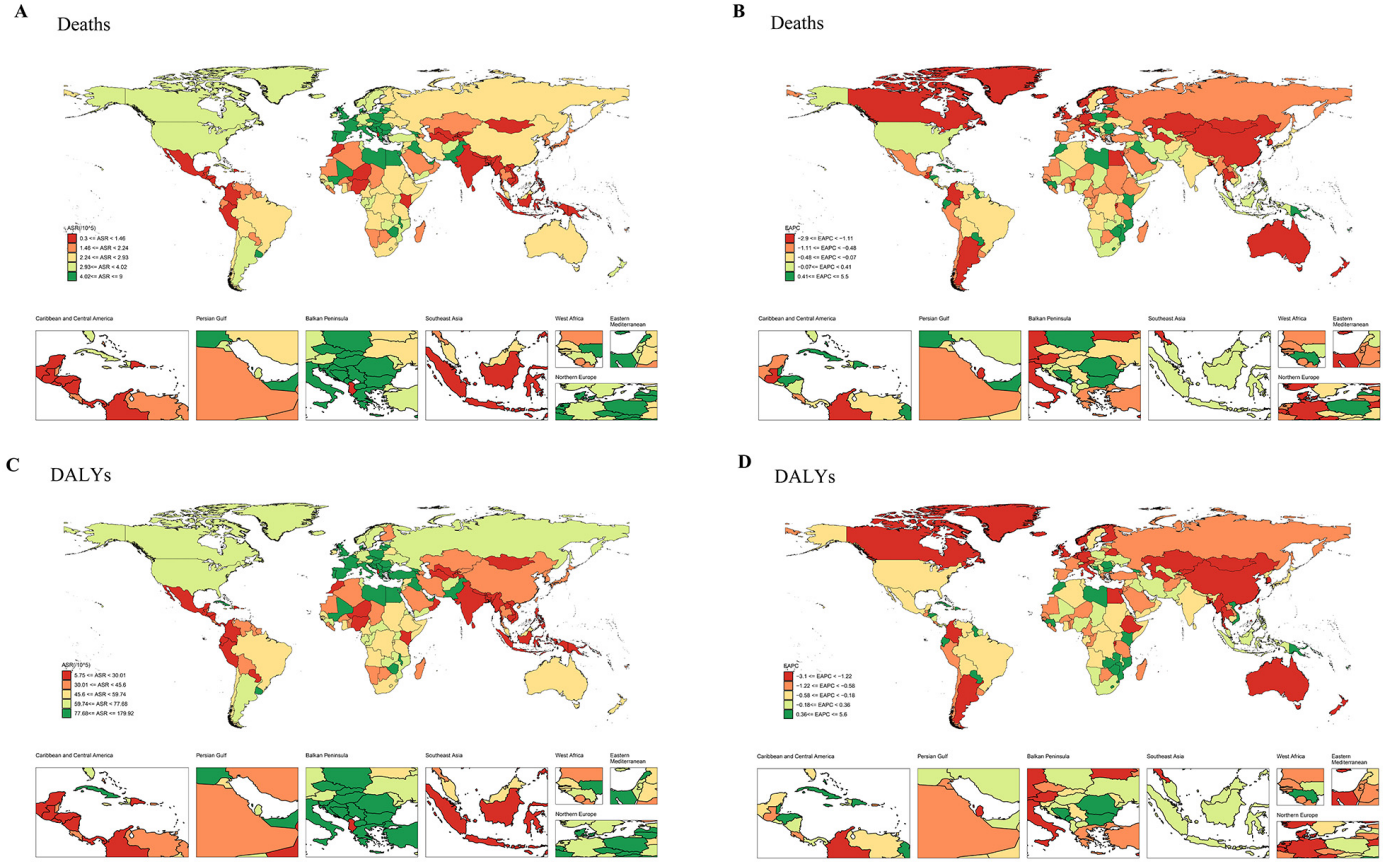
The ASR and EAPC maps of bladder cancer in 204 countries and territories. **(A)** ASR map for deaths. **(B)** EAPC map for deaths. **(C)** ASR map for DALYs. **(D)** EAPC map for DALYs. ASR, age-standardized rate; EAPC, estimated annual percentage change; DALYs, disability-adjusted life years.

The incidence of bladder cancer varies significantly across regions, with higher incidence generally observed in the high-income regions (5). Moreover, in 204 countries and territories worldwide, the 2021 Global Burden of Disease (GBD) study for bladder cancer is displayed in **Figure 1**; **Supplementary Table 1**. Data reveal that, in 2021, the age-standardized rate (ASR) of bladder cancer-related deaths (ASDR) is the highest in Mali, with an ASDR of 8.994 per 100,000 persons (95% uncertainty interval (UI): 6.956-11.522) and estimated annual percentage change (EAPC) of 0.07 (95% confidence interval (CI): 0.01 to 0.14) (**Figures 1A, B**; **Supplementary Table 1**). The disability-adjusted life years (DALYs) of bladder cancer are the highest in Malawi, with the age-standardized DALY rate of 179.915 per 100,000 persons (95% UI: 140.229-227.226) and EAPC of 0.29 (95% CI: 0.12 to 0.47) (**Figures 1C, D**; **Supplementary Table 1**). Notably, from 1990 to 2021, the increase in bladder cancer-deaths and DALYs are the most in Cabo Verde, indicating the high disease burden in this country (**Figure 1**; **Supplementary Table 1**). Another 2021 GBD study illustrates that the ASR of bladder cancer-related incidence exhibits an upward trend in the middle and low socio-demographic index (SDI) regions, with the highest age-standardized DALY rate in Central Europe (25). The significant variation in bladder cancer-related deaths and DALYs across countries may reflect substantial disparities in the healthcare systems and prevention strategies for bladder cancer.

The differences in bladder cancer incidence across different regions may be closely related to environmental factors, tobacco exposure, and other factors (8). These factors can influence the composition for the urinary microbiota, which in turn affects the formulation of disease intervention strategies. Furthermore, dietary habits, can have a profound effect on the urinary microbiome (26). Diet risk may alter the structure of the urinary microbiome, thereby influencing the development of bladder cancer (27). Therefore, when addressing bladder cancer patients in different regions, it is essential to consider local environment and microbiome characteristics to develop more targeted intervention strategies.

### 2.2 Differences in urinary microbiota between healthy individuals and bladder cancer patients

Compared to healthy individuals, there are significant differences in the urinary microbiome composition of patients with urogenital cancers. Lactobacillus is enriched in the urine of adult women and may be valuable in enhancing the response of Bacillus Calmette-Guerin treatment for bladder cancer (28). For instance, Lactobacillus helps regulate urinary pH, creating an acidic environment that inhibits the growth of harmful pathogenic bacteria. Lactobacillus has antimicrobial properties, which further protect the urinary tract from infections (29). Additionally, the immune system in healthy individuals effectively controls the population of these microorganisms, preventing overgrowth. However, significant differences exist between the urinary microbiota of bladder cancer patients and healthy individuals (30, 31) (**Table 1**). The urine of bladder cancer patients often contains higher levels of pathogenic microorganisms compared to healthy individuals, including *Escherichia coli*, *Klebsiella* spp., and intestinal anaerobes (30). In bladder cancer patients, the abundance of polycyclic aromatic hydrocarbons-degrading bacteria such as *Sphingomonas*, *Acinetobacter*, *Micrococcus*, *Pseudomonas* and *Ralstonia* are increased (31). These bacteria typically enter the urine via the urethra, but bladder cancer patients are unable to effectively eliminate these pathogens due to immune system suppression. Moreover, the urinary microbiota is further altered by antibiotic treatment and additional immunosuppression caused by chemotherapy/radiotherapy. Studies have shown a high prevalence of antibiotic-resistant bacteria in the urine of bladder cancer patients, particularly in those undergoing long-term treatment. These resistant bacteria include *Klebsiella oxytoca*, *Morganella morganii*, *Salmonella enterica*, and *Trabulsiella farmeri* (32). The colonization of these antibiotic-resistant bacteria not only increases the risk of infections but may also worsen disease prognosis.

**Table 1:** Characteristics of urinary microbiota between healthy individuals and bladder cancer patients.

Host	Population	Dominant bacteria	Author	Journal
Human	Total 708 Healthy individuals 259 Bladder cancer patients 449	*Escherichia coli*, *Klebsiella* spp. and intestinal anaerobes	Abdourahamane Yacouba et al. (30)	Seminars in Cancer Biology
Human	Total 189 Healthy individuals 60 Bladder cancer patients 129	Polycyclic aromatic hydrocarbon-degrading bacteria *Sphingomonas*, *Acinetobacter*, *Micrococcus*, *Pseudomonas* and *Ralstonia*	Laura Bukavina et al. (31)	European Urology Oncology

### 2.3 Bladder cancer subtypes and microbiota characteristics

Bladder cancer is classified into non-muscle invasive bladder cancer and muscle-invasive bladder cancer based on the depth of invasion into the bladder wall. These two subtypes of bladder cancer differ in clinical presentation, treatment approaches, and prognosis. Recent studies have suggested that the different subtypes of bladder cancer are also associated with variations in the urinary microbiota of patients (33). Non-muscle invasive bladder cancer is typically diagnosed at an earlier stage, with tumors confined to the epithelial layer of the bladder and not extending into the muscle layer. Since this type of cancer is diagnosed early, the immune system of the patient has not been severely compromised, and as a result, the urinary microbiota retains a certain level of diversity (34). In contrast, muscle-invasive bladder cancer, characterized by tumor invasion into the muscle layer of the bladder, places a greater burden on the patient’s immune system (35). A clinical study shows that the content of Cupriavidus in patients with NMIBC is increased significantly (36). Moreover, the proportions of Haemophilus and Veillonella in the urine of muscle-invasive bladder cancer patients are significantly higher than those in non-muscle invasive bladder cancer patients (36). This dysbiosis may be closely related to the immunosuppressive state of bladder cancer patients.

### 2.4 Possible mechanisms of dysbiosis

The dysbiosis of the urinary microbiota in bladder cancer patients is not incidental, but rather influenced by multiple factors, primarily including inflammation, immune suppression, and changes in metabolic products (37). Bladder cancer is a malignant tumor, and the onset and progression of the tumor lead to chronic inflammatory responses in the bladder. Research has shown that microbiota-induced inflammation is one of significant factors contributing to the deterioration caused by the bladder cancer (38). The persistent local inflammatory response in bladder cancer patients may provide a favorable growth environment for pathogenic microorganisms. Inflammation alters the pH, oxygen levels, and nutritional components of urine through the secretion of cytokines, recruitment of immune cells, and changes in the local microenvironment, thereby influencing the growth and colonization of microorganisms (39). Moreover, after undergoing treatments such as chemotherapy, radiotherapy, or cystectomy, the immune function of bladder cancer patients is significantly suppressed. The immunosuppressive state can further promote the immune escape of cancer cells (40). Additionally, the metabolic products within the bodies of bladder cancer patients can affect the composition of the urinary microbiota (41). The metabolic activity of cancer cells differs from that of normal cells. Lactate and other organic acids produced during tumor cell metabolism may create a favorable environment for the growth of anaerobic bacteria (42).

## 3 Dynamic changes of urinary microbiota in patients with bladder cancer after different surgeries

With the continuous advancement of surgical treatments, some complex issues faced by bladder cancer patients during their postoperative recovery process have gradually gained attention, especially the dynamic changes in the urinary microbiota following bladder cancer surgery and its relationship with patient recovery. In recent years, an increasing number of studies have shown that the structure, function, and diversity of the urinary microbiota undergo significant changes in bladder cancer patients postoperatively, which may be closely related to postoperative infections, immune system reconstruction, and disease recurrence (18). The urinary microbiota of bladder cancer patients experiences dynamic changes after surgery, and these changes are not only closely associated with the patient’s immune response, treatment methods (antibiotic use and chemotherapy), and postoperative complications, but also influence the patient’s postoperative recovery and survival prognosis to some extent. After bladder cancer surgery, patients may experience urinary tract infections, alterations in antibiotic-resistant bacterial populations, and dysbiosis of beneficial microbiota in the urine (43, 44).

### 3.1 Impact of surgical methods on urinary microbiota

The surgical management of bladder cancer primarily involves two key procedures: transurethral resection of bladder tumor and radical cystectomy (45). Transurethral resection of bladder tumor is typically used for non-muscle invasive bladder cancer, whereas radical cystectomy is often required for more advanced or muscle-invasive cases. Research has indicated that these different surgical approaches can significantly affect the urinary microbiota, with each method having distinct influences on the composition and diversity of microbial communities present in the urine (46, 47) (**Table 2**). After undergoing transurethral resection of bladder tumor, patients may experience structural shifts in their urinary microbiota. These changes can be attributed to various factors such as urethral injury during the resection process, the insertion of urinary catheters, and potential postoperative infections. These factors can introduce disturbances in the natural balance of the microbial environment in the urinary system, potentially leading to an altered microbiome (46). On the other hand, radical cystectomy, which involves the complete removal of the bladder, can lead to even more substantial alterations in the urinary microbiota (47) (**Table 3**). This is due to the complex procedures involved in urinary tract reconstruction following cystectomy. Different techniques for reconstructing the urinary tract, such as the creation of an ileal conduit or an ileal neobladder, can significantly impact the physiological characteristics of the urine. These changes may include variations in the pH level, the ionic composition, and other aspects of urine chemistry, all of which can affect the microbial ecosystem within the urinary system. Under normal conditions, the pH of urine ranges from 4.5 to 8.0. However, the pH of the ileum is typically alkaline. When using an ileal conduit or ileal neobladder, the mucosa of the ileum comes into contact with urine, leading to the increase of urine pH, but it is still acidic (48). Additionally, the storage of urine in intestinal mucosa will lead to the reabsorption of urea, potassium and chloride, and the excretion of sodium and bicarbonate, which will lead to the increase of acid load (49). Beyond pH and ionic composition, the level of urinary components may also change (48). The increase in ammonia in the urine may lead to a higher pH, further altering the ecosystem of the microbiome. As a result, the type of surgical procedure performed plays an important role in shaping the urinary microbiota, which may have implications for the patient’s recovery and overall health (46).

**Table 2:** Impact of surgical methods on urinary microbiota.

Surgical	Mean age	Gender	Application	Tumor stage	Main factors for the changes of urinary microbiota	Reference
Transurethral resection of bladder tumor	69.0	8 male 3 female	NMIBC	LG	Urethral injury during the resection process, the insertion of urinary catheters and potential postoperative infections	(46)
Radical cystectomy	63.6	70 male 38 female	MIBC	≤pT2 16 >pT2 33	Urinary tract reconstruction	(47)

NMIBC, non-muscle invasive bladder cancer; MIBC, muscle invasive bladder cancer; LG, low grade; pT, pathological T stage.

**Table 3:** Comparison of urinary microbiota profiles across patient subtypes: pre-operative vs. post-operative and NMIBC vs. MIBC.

Patients with bladder cancer	First author, year of publication	Tumor stage	Surgical method	Main findings
Pre-operative	Yacouba, 2022 (30)	/	No	In voided urine, *Acinetobacter*, Actinomyces, Aeromonas, Anaerococcus, *Pseudomonas*, and Tepidomonas were increased in the bladder cancer patients, while Lactobacillus, Roseomonas, Veillonella were increased in the control groups.
Post-operative	Ślusarczyk, 2024 (46)	NMIBC	Transurethral resection of bladder tumor	Postoperative urinary microbial phylogenetic alpha diversity was lower than preoperative levels. Actinomyces, Candidatus cloacimonas, Sphingobacterium, Sellimonas, Fusobacterium, and Roseobacter were more differentially enriched taxa in urine at the follow-up cystoscopy than at the index TURBT.
Post-operative	Pederzoli, 2020 (47)	MIBC ≤pT2 16 >pT2 33	Radical cystectomy	The genus *Klebsiella* was more common in the urine of female BCa patients versus healthy controls, while no clinically relevant bacteria were found differently enriched in men.

NMIBC, non-muscle invasive bladder cancer; MIBC, muscle invasive bladder cancer; pT, pathological T stage; TURBT, transurethral resection of bladder tumor.

### 3.2 Antibiotic use and changes in the urinary microbiota

Bladder cancer patients often require antibiotic therapy following surgical procedures to prevent or treat infections that may arise as a result of the surgery or subsequent postoperative complications. Antibiotics play an essential role in managing infections by targeting and eliminating harmful pathogens that could lead to more severe health issues. However, the prolonged or excessive use of antibiotics, especially broad-spectrum antibiotics, can have unintended consequences (50). One such consequence is the disruption of the natural balance of the urinary microbiota, a condition known as dysbiosis (51). Dysbiosis refers to an imbalance in the microbial community, where the beneficial bacteria that help maintain a healthy microbiome are reduced in number, while harmful or pathogenic bacteria proliferate. This imbalance can have serious consequences for the urinary tract health (52). The overuse of antibiotics can result in the overgrowth of pathogenic microorganisms such as *Escherichia coli* and *Klebsiella* spp., which are known to be harmful to the urinary tract. *Escherichia coli* may evade immune surveillance by altering the function of host immune cells. It can suppress T cell function by secreting immunosuppressive factors, thereby reducing the body’s immune response (53). Meanwhile, the populations of beneficial bacteria, like Lactobacillus and Bifidobacterium spp., that play a crucial role in supporting a healthy microbiome, are diminished. This microbial imbalance creates an environment more prone to infections, particularly urinary tract infections, which are common in bladder cancer patients, particularly those who have undergone surgical interventions (21). Infections caused by antibiotic-related dysbiosis may delay the healing process, impair immune function, and exacerbate other health issues, which can ultimately lead to poorer treatment outcomes (54). This highlights the importance of carefully managing antibiotic use, not only to treat infections but also to preserve the integrity of the urinary microbiota. By balancing the need for antibiotic intervention with efforts to minimize disruptions to the microbiota, clinicians can help ensure better recovery outcomes and reduce the risks associated with dysbiosis. Therefore, while antibiotics are a crucial component of infection management, close monitoring and appropriate prescribing practices are necessary to avoid their adverse effects on the patient’s recovery and long-term survival.

### 3.3 Changes in dominant microbial communities

The structure of the urinary microbiota in bladder cancer patients often experiences significant changes after surgery, with alterations in the dominant microbial communities and an increase in certain pathogenic bacteria (55). The dominant microbial communities refer to the bacterial species that occupy a substantial proportion of the microbiota in a particular environment. In bladder cancer patients, especially following surgery, the urinary microbiota undergoes changes (56). After transurethral resection of bladder tumor, the quantities of Virongella and Bifidobacterium in the urine of patients with bladder cancer are increased (56). Research has shown that after procedures like cystectomy, there is often an increase in the abundance of these pathogenic bacteria in the urine. This phenomenon is closely linked to the use of postoperative antibiotics and the trauma caused by surgery. These changes can contribute to urinary tract infections and other complications, further complicating the recovery process for bladder cancer patients (57).

### 3.4 Proliferation of pathogenic bacteria

Postoperatively, especially following radical cystectomy, bladder cancer patients may experience excessive proliferation of certain pathogenic bacteria. The most common pathogenic bacteria include *Escherichia coli*, Enterococcus spp., and Staphylococcus spp. (50). The overgrowth of these pathogenic bacteria can lead to postoperative urinary tract infections, urosepsis, and other complications, thereby affecting the patient’s recovery (57). Specifically, in patients following cystectomy, alterations in urine pH, ion concentration, and urinary flow dynamics may create a favorable environment for the growth of certain pathogenic bacteria.

### 3.5 Changes in urinary microbiota diversity

The diversity of the urinary microbiota is an important indicator of the health of microbial communities and is typically assessed through α-diversity and β-diversity (55). α-diversity reflects the species richness and evenness within a single environment, while β-diversity indicates the differences in microbial communities between different environments. A study has shown that the α-diversity of the urinary microbiota in bladder cancer patients typically decreases after transurethral resection of the bladder tumor (46). This reduction in diversity may increase the risk of urinary tract infections and impact the patient’s postoperative immune recovery. The β-diversity of the urinary microbiota in bladder cancer patients postoperatively generally shows significant variation. An increase in β-diversity indicates greater differences in the urinary microbiota between different patients (46). After transurethral resection of bladder tumor, the β-diversity of the urinary microbiota in bladder cancer patients changes significantly over time (46). This variation may be closely related to the physiological changes brought about by postoperative urinary tract reconstruction.

### 3.6 Functional changes in the urinary microbiota

The functionality of the urinary microbiota in bladder cancer patients also undergoes changes after Bacillus Calmette-Guerin treatment, particularly in terms of metabolic functions and resistance (32). These functional changes in the urinary microbiota can directly influence the occurrence of postoperative infections, the recovery of the patient’s immune function, and cancer recurrence. Changes in the metabolic functions of the urinary microbiota post-surgery may affect the patient’s postoperative recovery. One key area of research in bladder cancer postoperative care is the immune-regulatory role of the urinary microbiota. The urinary microbiota interacts with the host immune system, influencing the immune response (58). Beneficial bacteria, such as Lactobacillus, help maintain immune balance in the urine through mechanisms like promoting immune cell activation, increasing the secretion of anti-inflammatory factors, and suppressing inflammatory responses. However, dysbiosis in the urinary microbiota may lead to immune dysfunction, which can negatively affect the patient’s recovery and contribute to immune escape phenomena (58). With the widespread use of antibiotics, bladder cancer patients may face the challenge of antibiotic-resistant bacterial populations postoperatively. Recent studies have shown that the dysbacteriosis caused by antibiotic treatment will increase the risk of failure of immunoscreen inhibitor treatment (59). The emergence of antibiotic-resistant bacteria complicates treatment regimens, increases the risk of infections, and adversely affects the patient’s survival prognosis.

### 3.7 Urinary microbiota characteristics related to postoperative infections

Bladder cancer patients often face a range of complications following surgical treatment, with postoperative infections, particularly urinary tract infections, being among the most common issues (43, 44). In some cases, urinary tract infections are not merely short-term clinical manifestations; they can also lead to the colonization of antibiotic-resistant bacteria, further complicating and making treatment more difficult. The occurrence of urinary tract infections may stem from either postoperative infections or pre-existing chronic bacterial infections, especially in immunocompromised patients (58). Moreover, bladder cancer patients often receive broad-spectrum antibiotics postoperatively, which, while effectively preventing infections, may also lead to dysbiosis of the normal microbiota, providing an opportunity for the proliferation of resistant bacteria (32). The occurrence of postoperative infections not only impacts the patient’s recovery process but can also increase hospitalization time, treatment costs, and in some cases, lead to the complications of urinary tract infections and surgical site infections (60). The presence of these resistant bacteria may affect the patient’s prognosis and treatment strategies.

The prolonged use of antibiotics severely impacts the normal microbiota in the urine, inhibiting the growth of beneficial bacteria while selectively promoting the proliferation of resistant bacteria, leading to the colonization of resistant strains. When bladder cancer patients receive postoperative antibiotic treatment, bacteria that were originally sensitive to antibiotics may be suppressed, while resistant bacteria survive and establish colonization due to their resistance (44). One study found that, in patients undergoing radical cystectomy, the bacterial pathogens in greatest proportions are Enterococcus (42.0%), *Escherichia coli* (21.70%), and Candida (13.0%) (61). Additionally, bladder cancer patients often require the insertion of a urinary catheter postoperatively to maintain urinary tract patency. Prolonged catheterization can lead to mechanical irritation of the urethra, increasing the risk of urinary tract infections, particularly when catheters are not replaced in a timely manner (62). The presence of a catheter provides a habitat for bacterial colonization, thereby facilitating the invasion of antibiotic-resistant bacteria. A study has shown that the cultures from discharge catheters are closely associated with postoperative urinary tract infections (63). Therefore, timely replacement of catheters and strict adherence to sterile techniques are critical to reducing colonization by antibiotic-resistant bacteria.

### 3.8 Association between urinary microbiota and LUTS and recurrence

Changes in the urinary microbiome are closely related to patients’ lower urinary tract symptoms (LUTS). A spearman’s correlation analysis revealed that urine samples showing the presence of the bacterial genera Haemophilus, Staphylococcus, Listeria, Dolosigranulum, Phascolarctobacterium, Enhydrobacter, Bacillus, [Ruminococcus] torques, Faecalibacterium, and Finegoldia correlated with a high international prostate symptom score (IPSS), and severe storage and voiding symptoms (64). A study showed that compared with patients with primary bladder cancer, patients with recurrent bladder cancer had lower diversity in their urinary microbiota or were dominated by specific microbial groups. The relative abundance of Firmicutes was significantly higher and Bacteroidetes significantly lower in patients with recurrent bladder cancer (34).

## 4 Characteristics of the urinary microbiota after ileal neobladder reconstruction

Ileal neobladder reconstruction is a common urinary tract reconstruction surgery performed after bladder cancer surgery. It is typically used following radical cystectomy, where a portion of the ileum is utilized to replace the function of the bladder (65). In ileal neobladder reconstruction, part of the ileum is reconfigured into a urinary pouch and connected to the urethra, allowing patients to void. Postoperatively, patients typically need to urinate through the urethra, an artificial urethra, or a catheter (65). Ileal neobladder reconstruction is associated with a high rate of improvement in quality of life and urinary function recovery, making it an effective treatment option for bladder cancer patients (66, 67). However, the risk of urinary tract infections increases following ileal neobladder reconstruction, and changes in the urinary microbiota play an important role in postoperative recovery. Recent studies have demonstrated that ileal neobladder reconstruction not only alters the histological architecture but also causes substantial modifications of both the compositional profile and functional dynamics of the urinary microbiota (57).

### 4.1 Changes in the urinary microbiota after ileal neobladder reconstruction

The urinary microbiota refers to the various bacteria, fungi, and other microorganisms present in the urine (68). Under normal conditions, urine is considered sterile; however, because ileal neobladder reconstruction involves connecting intestinal tissue to the urinary system, different microbial communities may be present in the urine postoperatively. Recent studies have shown that the characteristics of the urinary microbiota in bladder cancer patients undergoing ileal neobladder reconstruction undergo significant changes (57). Due to the incorporation of the ileum, intestinal microbiota may colonize the urine, including species such as *Escherichia coli*, *Klebsiella* spp., and Enterococcus spp. These microbiota differ markedly from the microbiota typically found in the urine of healthy individuals (57). Additionally, endogenous bacteria from the ileal tissue may interact with the external environment through the urinary tract, leading to dysbiosis of the urinary microbiota. The incidence of urinary tract infections is higher in bladder cancer patients following ileal neobladder reconstruction (69). The colonization of antibiotic-resistant bacteria in the urine is one of the primary causes by postoperative infections. Research has shown that following ileal neobladder reconstruction, the urine of patients contains higher levels of E. coli, *Klebsiella* spp., Enterococcus spp., and other bacteria with higher resistance to commonly used antibiotics such as amoxicillin and ampicillin (70). The colonization by these antibiotic-resistant bacteria may lead to chronic urinary tract infections, and their resistance to traditional antibiotic treatments complicates the treatment process.

### 4.2 Clinical significance of ileal neobladder reconstruction on the urinary microbiota of bladder cancer patients

The impact of ileal neobladder reconstruction on the urinary microbiota of bladder cancer patients holds significant clinical implications. Firstly, this procedure alters the composition of the urinary microbiota, potentially leading to the colonization by antibiotic-resistant bacteria and an increased risk of urinary tract infections (69). Secondly, postoperative infections may result in extended hospitalization, increased treatment costs, and could even affect the patient’s long-term survival prognosis. Therefore, understanding the effects of ileal neobladder reconstruction on the urinary microbiota and implementing appropriate infection prevention and treatment measures are crucial for improving patient outcomes. To reduce the occurrence of urinary tract infections after ileal neobladder reconstruction, patients should undergo strict infection control protocols. Tailoring personalized antibiotic treatment plans based on the changes in the urinary microbiota post-surgery is essential. Additionally, as patients undergoing this procedure often have weakened immune systems, enhancing immune function regulation is also critical for infection prevention. Further research has revealed that the occurrence of LUTS in patients after ileal neobladder surgery is closely related to specific changes in the microbiota. Kim et al. found that Enterococcus was significantly enriched in patients with postoperative febrile urinary tract infections, and these patients had an increased incidence of urinary urgency and nocturia (57). So, microbiota-targeted interventions may offer novel therapeutic strategies for preventing recurrence and improving patient outcomes.

## 5 Research prospects

The study of urinary microbiota as a biomarker for prognostic evaluation in bladder cancer patients has emerged as a growing focus in the field of oncology (71). With the continuous development of microbiomics, research on the urinary microbiota offers new directions for cancer diagnosis, prognostic assessment, and treatment strategies (23). Bladder cancer, as a common urological malignancy, has long relied on traditional imaging techniques and histopathological examinations for treatment and prognostic evaluation. However, with the continuous advancements in molecular biology techniques, an increasing number of studies have shown that the urinary microbiota not only plays a significant role in the occurrence and development of bladder cancer but can also serve as a non-invasive biomarker, providing strong support for early diagnosis, prognostic evaluation, and treatment of bladder cancer.

### 5.1 Relationship between the urinary microbiota and bladder cancer

Recent studies have shown that the development of bladder cancer is influenced not only by environmental factors, genetic background, and lifestyle but also by dysbiosis of the local bladder microbiota (22, 23). As an important organ of the urinary system, the bladder is in prolonged contact with microorganisms in urine, and changes in the urinary microbiota may directly or indirectly affect the immune environment of the bladder, tumor-related signaling pathways, and the growth and metastasis of cancer cells (72). Research has found significant differences in the urinary microbiota composition between bladder cancer patients and healthy individuals (73). Specific bacterial communities, such as Akkermansia and Bacteroidetes, are often found in the urine of bladder cancer patients, with notable changes in their abundance and diversity (74). In addition to the early onset of bladder cancer, the urinary microbiota is also associated with cancer progression and metastasis. A study has shown that certain bacterial communities may promote tumor growth, invasion, and metastasis in the bladder cancer microenvironment (35). Therefore, the urinary microbiota not only holds potential diagnostic value in the early stages of bladder cancer but also plays a crucial role in the progression of the disease.

### 5.2 Potential of the urinary microbiota as a prognostic biomarker for bladder cancer patients

The urinary microbiota holds significant potential as a biomarker for the prognostic evaluation of bladder cancer. Traditional methods for assessing bladder cancer prognosis primarily rely on tumor staging, histological grading, imaging studies, and blood tests (75). However, these methods often have certain limitations and may not accurately reflect a patient’s actual prognosis. In contrast, the urinary microbiota offers advantages such as non-invasiveness, ease of collection, and high-throughput detection, providing valuable information for early screening, disease monitoring, and prognostic assessment in bladder cancer. A decrease in the diversity and abundance of the urinary microbiota may be associated with higher risk, higher recurrence rates, and lower survival rates in bladder cancer (76). Additionally, the abundance of Veillonella, Brevundimonas, and Methylobacterium genera in the urine of bladder cancer patients is significantly elevated, and changes in the abundance of these bacteria are closely linked to clinical staging, pathological grading, and the patient’s survival time (76). Therefore, the diversity and composition of the urinary microbiota may serve as important indicators for prognostic assessment in bladder cancer patients. Moreover, the high recurrence rate of bladder cancer is a critical factor impacting patient quality of life and prognosis. Research has found that the increase in certain pathogenic bacteria (Veillonella and Bifidobacterium) in urine may lead to the recurrence of bladder cancer (56). Thus, the urinary microbiota could serve as an early warning signal, helping to identify high-risk bladder cancer patients and providing a basis for early intervention in recurrence.

### 5.3 Future research directions

Although preliminary research on the relationship between the urinary microbiota and bladder cancer has yielded important initial findings, many unanswered questions remain. Therefore, future research should delve deeper in the following directions:

#### 5.3.1 Multi-omics integration analysis

Changes in the urinary microbiota are increasingly being recognized as potentially important factors in various diseases, including bladder cancer (30). These changes may be influenced by several biological processes, including the body’s metabolic and genetic pathways. In particular, factors such as metabolomics and transcriptomics are thought to play a significant role in shaping the urinary microbiota and influencing its impact on the health (77). Metabolomics involves the study of metabolites, the small molecules produced during metabolism, while transcriptomics focuses on gene expression profiles, revealing how genes are activated or deactivated in response to various biological conditions. Together, these fields offer deep insights into how changes in the urinary microbiota might contribute to disease processes like bladder cancer. To further explore how the microbiota interacts with other biological systems, future research could aim to examine the relationships between the urinary microbiota, the metabolome, and the transcriptome through the integration of multi-omics data. Multi-omics integration analysis allows researchers to gather data from various omics layers, such as metagenomics, which studies the microbial communities, metabolomics, and transcriptomics. By combining these techniques, researchers can generate a more holistic understanding of how the microbiota, metabolites, and gene expression patterns are interconnected and how they contribute to bladder cancer.

#### 5.3.2 Targeted microbiota intervention strategies

The urinary microbiota has emerged as a potential biomarker for the prognostic assessment of bladder cancer, offering new opportunities for personalized treatment approaches (22). As research into the role of the microbiota in various diseases advances, its significance in bladder cancer prognosis is becoming increasingly recognized. The unique microbial composition of the urinary tract could provide valuable insights into the course of the disease and help predict patient outcomes. Moreover, the urinary microbiota could serve as a new target for therapeutic intervention, opening up novel avenues for treatment that focus on modulating the microbial environment. These strategies, which include probiotics, are gaining popularity as potential methods to improve patient outcomes by influencing the microbial communities within the body (27, 78). Probiotics, which are live beneficial microorganisms, have the potential to restore a healthy balance between the intestinal microbiota and the urinary microbiota. By reintroducing beneficial bacteria, probiotics could help to modulate the immune system, potentially enhancing the body’s ability to fight bladder cancer (78).

Looking ahead, future research should focus on understanding how targeted modulation of the urinary microbiota could influence the development and progression of bladder cancer. By investigating the effects of probiotics and other microbiota-targeted interventions, scientists could uncover new ways to enhance cancer treatment and improve patient prognosis. Additionally, studies could explore the potential for personalized therapy based on the unique microbiota composition of each patient, offering a more tailored and effective approach to bladder cancer management. Further research is needed to explore the relationship between urinary microbiota and LUTS as well as cancer recurrence, and to establish a predictive model that includes microbial parameters, LUTS scores, and recurrence indicators. Ultimately, these innovative strategies could significantly improve treatment outcomes and the quality of life for bladder cancer patients, leading to better long-term survival rates and enhanced overall health.

#### 5.3.3 Clinical translation challenges

Although the urinary microbiota has shown considerable promise as a potential biomarker for prognostic evaluation in bladder cancer, its successful clinical application is still hindered by a number of challenges. One of the primary obstacles is the unclear causal relationship between the urinary microbiota and the development of bladder cancer. While several studies have suggested a potential association, the exact mechanisms through which the microbiota may influence cancer progression are not well understood. To address this gap, more extensive and carefully designed prospective studies are needed to establish whether there is a direct causative link between changes in the urinary microbiota and the onset or progression of bladder cancer. Additionally, designing well-structured, large-scale clinical trials to assess the accuracy, reliability, and overall clinical utility of the urinary microbiota as a prognostic biomarker remains a critical issue. These trials must account for various factors such as patient demographics, disease stages, and treatment regimens, which could impact the microbiota. Moreover, the standardization of methods for analyzing and interpreting urinary microbiota data is essential for ensuring consistency across different studies. Overcoming these challenges will be key to unlocking the full potential of the urinary microbiota as a useful tool for predicting outcomes and guiding treatment decisions in bladder cancer patients.

## 6 Conclusion

In conclusion, this article reviewed the dynamic changes in the urinary microbiota of bladder cancer patients after surgery and their clinical significance. It summarized the role of different urinary microbiota in the postoperative recovery process of the disease and their impact on patient prognosis, while also discussing the challenges and limitations present in current research. It is hoped that further research on the changes in the urinary microbiota will provide new clinical insights for postoperative management of bladder cancer and lay the foundation for deeper exploration and innovative development in this field in the future.
