# Supplementary material for: Dynamic changes of urinary microbiota in patients with bladder cancer after surgery and its clinical significance
Source: Front Immunol. 2025 Sep 1;16:1638628. doi: 10.3389/fimmu.2025.1638628 (PMC12434072; doi:10.3389/fimmu.2025.1638628)
Supplement: Supplementary file 1 [file Table1.docx]

**Supplementary Table 1 The global disease burden of bladder cancer in 204 countries and territories.**

| **Location name** | **1990** | | **2021** | | **EAPC (95% CI)** |
| --- | --- | --- | --- | --- | --- |
|  | **Number** | **ASR (95% UI)** | **Number** | **ASR (95% UI)** |  |
| Deaths |  |  |  |  |  |
| Afghanistan | 250 (155-356) | 4.148 (2.785-5.836) | 288 (178-390) | 3.666 (2.412-4.828) | -0.47 (-0.52 to -0.43) |
| Albania | 6 (5-7) | 0.333 (0.265-0.419) | 13 (10-18) | 0.305 (0.228-0.417) | -0.01 (-0.16 to 0.15) |
| Algeria | 171 (124-213) | 2.16 (1.496-2.721) | 458 (350-584) | 1.787 (1.378-2.268) | -0.23 (-0.45 to 0) |
| American Samoa | 0 (0-0) | 1.286 (1.071-1.731) | 1 (1-1) | 1.943 (1.623-2.309) | 2.17 (1.77 to 2.56) |
| Andorra | 3 (2-5) | 6.136 (4.308-8.709) | 6 (4-9) | 3.835 (2.601-5.201) | -1.34 (-1.6 to -1.08) |
| Angola | 81 (60-110) | 2.707 (2.063-3.614) | 227 (170-301) | 2.564 (1.946-3.357) | -0.16 (-0.24 to -0.07) |
| Antigua and Barbuda | 2 (1-2) | 2.696 (2.481-2.899) | 3 (2-3) | 2.767 (2.54-2.969) | 0.43 (0.17 to 0.69) |
| Argentina | 1529 (1426-1622) | 4.901 (4.571-5.186) | 1718 (1576-1868) | 2.964 (2.725-3.219) | -1.38 (-1.48 to -1.27) |
| Armenia | 122 (104-144) | 4.788 (4.072-5.64) | 224 (187-265) | 5.134 (4.276-6.057) | 0.17 (-0.06 to 0.41) |
| Australia | 762 (711-809) | 3.916 (3.634-4.157) | 1399 (1224-1548) | 2.719 (2.4-2.99) | -1.15 (-1.23 to -1.07) |
| Austria | 605 (562-641) | 4.78 (4.424-5.057) | 679 (593-742) | 3.219 (2.832-3.501) | -1.12 (-1.23 to -1.01) |
| Azerbaijan | 96 (75-128) | 2.011 (1.573-2.661) | 163 (118-223) | 1.759 (1.296-2.422) | -0.29 (-0.43 to -0.14) |
| Bahamas | 2 (2-3) | 1.635 (1.51-1.77) | 7 (5-8) | 1.819 (1.502-2.202) | 0.66 (0.53 to 0.79) |
| Bahrain | 10 (8-12) | 8.073 (6.444-10.097) | 28 (20-39) | 5.127 (3.807-6.898) | -1.94 (-2.22 to -1.66) |
| Bangladesh | 613 (427-927) | 1.489 (1.028-2.255) | 1394 (849-2652) | 1.152 (0.715-2.21) | -1.11 (-1.35 to -0.88) |
| Barbados | 8 (7-8) | 2.474 (2.296-2.648) | 14 (11-16) | 2.579 (2.082-3.11) | 0.44 (0.27 to 0.61) |
| Belarus | 425 (391-459) | 3.294 (3.043-3.549) | 409 (333-484) | 2.476 (2.033-2.943) | -1.68 (-2.09 to -1.27) |
| Belgium | 1042 (958-1107) | 6.473 (5.978-6.87) | 1131 (974-1253) | 4.139 (3.634-4.555) | -1.31 (-1.54 to -1.09) |
| Belize | 1 (1-2) | 1.649 (1.518-1.769) | 5 (5-6) | 1.98 (1.721-2.231) | 0.83 (0.4 to 1.25) |
| Benin | 43 (34-54) | 2.411 (1.91-3.032) | 79 (62-103) | 1.836 (1.448-2.378) | -1.14 (-1.27 to -1) |
| Bermuda | 4 (4-4) | 6.828 (6.298-7.333) | 7 (6-8) | 4.514 (3.833-5.401) | -0.82 (-1.08 to -0.56) |
| Bhutan | 2 (1-4) | 1.231 (0.772-1.975) | 6 (4-13) | 1.176 (0.769-2.426) | -0.21 (-0.3 to -0.12) |
| Bolivia (Plurinational State of) | 67 (48-88) | 2.49 (1.786-3.247) | 178 (129-247) | 2.262 (1.65-3.117) | -0.25 (-0.3 to -0.21) |
| Bosnia and Herzegovina | 116 (99-136) | 3.155 (2.705-3.727) | 269 (200-340) | 4.127 (3.072-5.209) | 1.1 (0.96 to 1.25) |
| Botswana | 11 (9-14) | 2.519 (1.939-3.114) | 25 (20-34) | 2.07 (1.667-2.723) | -0.79 (-0.96 to -0.62) |
| Brazil | 2029 (1919-2115) | 2.661 (2.487-2.781) | 5819 (5279-6165) | 2.402 (2.173-2.549) | -0.22 (-0.28 to -0.17) |
| Brunei Darussalam | 3 (2-3) | 3.365 (2.717-4.12) | 6 (5-7) | 2.507 (2.044-3.033) | -0.15 (-0.43 to 0.13) |
| Bulgaria | 452 (400-502) | 4.03 (3.614-4.411) | 778 (666-918) | 5.171 (4.405-6.111) | 1.25 (1.12 to 1.38) |
| Burkina Faso | 85 (63-112) | 2.407 (1.815-3.17) | 147 (113-204) | 1.904 (1.473-2.585) | -0.98 (-1.12 to -0.84) |
| Burundi | 67 (51-85) | 3.286 (2.528-4.138) | 93 (69-126) | 2.464 (1.844-3.273) | -1.3 (-1.44 to -1.15) |
| Cabo Verde | 1 (0-2) | 0.258 (0.173-0.638) | 9 (4-11) | 2.04 (1.078-2.713) | 5.4 (3.94 to 6.88) |
| Cambodia | 56 (44-74) | 1.482 (1.159-1.952) | 146 (105-208) | 1.395 (0.994-2.012) | -0.24 (-0.44 to -0.05) |
| Cameroon | 99 (81-119) | 2.801 (2.334-3.335) | 247 (188-338) | 2.419 (1.883-3.261) | -0.73 (-0.87 to -0.59) |
| Canada | 1697 (1558-1800) | 5.184 (4.755-5.504) | 2728 (2399-3001) | 3.369 (2.986-3.691) | -1.33 (-1.46 to -1.2) |
| Central African Republic | 28 (21-37) | 3.155 (2.517-4.013) | 46 (33-62) | 2.737 (2.109-3.478) | -0.48 (-0.52 to -0.44) |
| Chad | 51 (39-65) | 2.037 (1.569-2.584) | 104 (77-136) | 2.209 (1.65-2.902) | 0.01 (-0.16 to 0.18) |
| Chile | 294 (276-311) | 3.173 (2.971-3.36) | 634 (572-685) | 2.407 (2.179-2.601) | -0.66 (-0.78 to -0.53) |
| China | 22765 (16777-26478) | 3.442 (2.577-3.944) | 45114 (36263-57335) | 2.336 (1.891-2.941) | -1.6 (-1.73 to -1.46) |
| Colombia | 311 (291-330) | 2.015 (1.884-2.145) | 779 (648-915) | 1.412 (1.172-1.659) | -1.45 (-1.64 to -1.25) |
| Comoros | 4 (3-5) | 2.429 (1.767-3.236) | 9 (6-14) | 2.288 (1.544-3.334) | -0.38 (-0.48 to -0.29) |
| Congo | 30 (21-41) | 3.514 (2.741-4.581) | 63 (47-83) | 3.1 (2.338-3.996) | -0.56 (-0.71 to -0.41) |
| Cook Islands | 0 (0-0) | 3.29 (2.758-3.86) | 1 (1-1) | 2.992 (2.378-3.692) | -0.16 (-0.27 to -0.06) |
| Costa Rica | 38 (35-41) | 2.31 (2.129-2.49) | 106 (91-119) | 1.937 (1.665-2.17) | -0.8 (-0.96 to -0.64) |
| Croatia | 294 (261-325) | 5.317 (4.747-5.856) | 526 (445-618) | 5.324 (4.539-6.225) | 0.25 (0.07 to 0.43) |
| Cuba | 379 (352-403) | 3.804 (3.545-4.033) | 829 (709-947) | 4.019 (3.442-4.592) | 0.45 (0.28 to 0.62) |
| Cyprus | 45 (36-57) | 7.617 (6.091-9.738) | 86 (67-109) | 4.249 (3.337-5.331) | -1.84 (-1.96 to -1.72) |
| Czechia | 733 (657-818) | 5.216 (4.703-5.8) | 1110 (935-1300) | 4.74 (3.979-5.574) | -0.38 (-0.49 to -0.26) |
| Democratic People's Republic of Korea | 281 (206-365) | 2.065 (1.527-2.653) | 549 (426-815) | 1.748 (1.365-2.612) | -0.45 (-0.52 to -0.38) |
| Democratic Republic of the Congo | 327 (251-420) | 2.829 (2.139-3.714) | 738 (536-987) | 2.597 (1.87-3.485) | -0.31 (-0.5 to -0.12) |
| Denmark | 444 (380-537) | 5.176 (4.461-6.211) | 609 (542-670) | 4.587 (4.103-5.01) | -1.33 (-1.9 to -0.77) |
| Djibouti | 3 (2-4) | 2.664 (2.002-3.576) | 13 (9-19) | 2.794 (1.981-3.971) | 0.06 (-0.01 to 0.12) |
| Dominica | 2 (2-2) | 3.547 (2.958-4.137) | 3 (2-3) | 3.525 (2.906-4.248) | 0.12 (0.06 to 0.19) |
| Dominican Republic | 35 (28-41) | 1.129 (0.906-1.363) | 111 (83-149) | 1.15 (0.866-1.544) | 0.47 (0.28 to 0.66) |
| Ecuador | 63 (58-69) | 1.361 (1.243-1.489) | 236 (188-295) | 1.536 (1.232-1.904) | 0.53 (0.24 to 0.81) |
| Egypt | 1922 (1312-2263) | 8.225 (6.526-9.647) | 2130 (1528-3546) | 4.395 (3.126-7.532) | -2.49 (-2.75 to -2.23) |
| El Salvador | 25 (21-28) | 0.867 (0.748-0.992) | 61 (49-75) | 0.94 (0.757-1.164) | 0.23 (0.13 to 0.33) |
| Equatorial Guinea | 5 (3-6) | 2.988 (2.311-3.888) | 11 (8-16) | 2.893 (2.056-3.96) | -0.01 (-0.22 to 0.2) |
| Eritrea | 24 (17-33) | 2.91 (2.115-3.81) | 61 (45-81) | 2.929 (2.237-3.881) | -0.07 (-0.13 to 0) |
| Estonia | 81 (74-88) | 3.957 (3.612-4.321) | 113 (98-130) | 3.671 (3.178-4.21) | -0.78 (-1 to -0.56) |
| Eswatini | 7 (5-8) | 2.705 (2.085-3.486) | 13 (9-18) | 2.726 (1.897-3.681) | 0.36 (0.01 to 0.72) |
| Ethiopia | 498 (388-677) | 3.171 (2.43-4.198) | 886 (630-1264) | 2.468 (1.766-3.509) | -1.06 (-1.2 to -0.92) |
| Fiji | 4 (3-5) | 1.346 (0.993-1.664) | 11 (7-15) | 1.726 (1.095-2.256) | 1.1 (0.95 to 1.26) |
| Finland | 283 (259-303) | 3.829 (3.505-4.101) | 342 (300-379) | 2.297 (2.044-2.52) | -1.89 (-2.01 to -1.76) |
| France | 5493 (5097-5832) | 6.238 (5.804-6.613) | 7752 (6729-8666) | 4.582 (4.037-5.125) | -0.9 (-0.94 to -0.87) |
| Gabon | 19 (15-24) | 3.721 (2.937-4.741) | 29 (21-39) | 3.367 (2.549-4.478) | -0.43 (-0.49 to -0.37) |
| Gambia | 4 (3-5) | 1.361 (1.039-1.71) | 12 (9-16) | 1.464 (1.067-1.874) | 0.13 (0.04 to 0.23) |
| Georgia | 229 (185-271) | 3.705 (3-4.384) | 312 (272-356) | 5.104 (4.417-5.819) | 1.13 (0.51 to 1.75) |
| Germany | 7210 (6660-7694) | 5.339 (4.941-5.68) | 8073 (7124-8779) | 3.526 (3.169-3.803) | -1.52 (-1.73 to -1.31) |
| Ghana | 132 (101-169) | 2.629 (2.051-3.328) | 330 (246-441) | 2.379 (1.765-3.232) | -0.45 (-0.56 to -0.33) |
| Greece | 1182 (1094-1250) | 7.717 (7.135-8.158) | 1741 (1549-1875) | 5.974 (5.433-6.423) | -1.01 (-1.2 to -0.83) |
| Greenland | 1 (1-2) | 5.52 (3.791-6.472) | 2 (1-3) | 3.556 (2.517-4.623) | -1.21 (-1.39 to -1.03) |
| Grenada | 2 (2-2) | 2.561 (2.301-2.837) | 3 (2-3) | 2.783 (2.369-3.135) | 0.53 (0.14 to 0.92) |
| Guam | 1 (1-1) | 1.323 (1.097-1.663) | 2 (2-3) | 1.182 (0.986-1.386) | 0.8 (0.44 to 1.17) |
| Guatemala | 30 (28-31) | 1.128 (1.072-1.181) | 78 (67-90) | 0.771 (0.67-0.881) | -1.41 (-1.64 to -1.18) |
| Guinea | 90 (69-111) | 3.095 (2.414-3.828) | 167 (126-226) | 3.486 (2.624-4.652) | 0.48 (0.42 to 0.54) |
| Guinea-Bissau | 10 (7-14) | 3.198 (2.249-4.198) | 15 (11-20) | 2.586 (1.958-3.345) | -0.84 (-0.93 to -0.75) |
| Guyana | 6 (5-6) | 1.712 (1.523-1.906) | 10 (7-12) | 1.684 (1.317-2.119) | 0.52 (0.33 to 0.72) |
| Haiti | 82 (56-109) | 3.115 (2.254-4.042) | 169 (120-228) | 2.983 (2.172-3.981) | 0.04 (-0.02 to 0.1) |
| Honduras | 9 (7-11) | 0.504 (0.405-0.614) | 40 (29-53) | 0.72 (0.523-0.963) | 1.39 (1.25 to 1.54) |
| Hungary | 799 (720-878) | 5.437 (4.901-5.969) | 1086 (939-1253) | 5.249 (4.546-6.047) | -0.15 (-0.39 to 0.09) |
| Iceland | 15 (13-16) | 4.938 (4.486-5.402) | 21 (18-24) | 3.255 (2.784-3.68) | -1.27 (-1.42 to -1.12) |
| India | 4570 (3560-5442) | 1.21 (0.945-1.448) | 12904 (11227-15142) | 1.23 (1.074-1.437) | -0.15 (-0.32 to 0.01) |
| Indonesia | 914 (729-1201) | 1.122 (0.872-1.516) | 2284 (1536-3824) | 1.189 (0.784-2.023) | 0.14 (0.12 to 0.17) |
| Iran (Islamic Republic of) | 480 (389-582) | 2.379 (1.932-2.862) | 1528 (1263-1728) | 2.242 (1.851-2.534) | -0.03 (-0.12 to 0.06) |
| Iraq | 323 (243-422) | 4.406 (3.308-5.782) | 1022 (738-1369) | 5.421 (3.939-7.248) | 0.43 (0.27 to 0.6) |
| Ireland | 185 (173-198) | 4.506 (4.188-4.815) | 258 (223-290) | 3.034 (2.631-3.401) | -1.11 (-1.3 to -0.93) |
| Israel | 229 (210-247) | 4.839 (4.422-5.195) | 518 (440-569) | 3.824 (3.278-4.203) | -0.95 (-1.2 to -0.69) |
| Italy | 6551 (6140-6823) | 7.103 (6.632-7.41) | 8725 (7606-9485) | 4.849 (4.318-5.242) | -1.27 (-1.33 to -1.2) |
| Jamaica | 46 (42-50) | 2.466 (2.254-2.675) | 81 (62-101) | 2.528 (1.954-3.191) | 0.41 (0.12 to 0.7) |
| Japan | 3925 (3610-4090) | 2.453 (2.233-2.565) | 11470 (9427-12614) | 2.223 (1.91-2.399) | -0.25 (-0.32 to -0.18) |
| Jordan | 35 (28-44) | 3.36 (2.636-4.196) | 151 (109-204) | 2.597 (1.926-3.456) | -0.99 (-1.12 to -0.86) |
| Kazakhstan | 293 (228-384) | 2.426 (1.894-3.161) | 323 (269-381) | 1.961 (1.641-2.301) | -1.3 (-1.78 to -0.83) |
| Kenya | 66 (49-83) | 0.962 (0.714-1.215) | 228 (184-274) | 1.26 (1.02-1.504) | 1.09 (0.97 to 1.22) |
| Kiribati | 0 (0-0) | 0.505 (0.399-0.612) | 0 (0-0) | 0.579 (0.432-0.716) | 0.49 (0.41 to 0.57) |
| Kuwait | 12 (11-13) | 2.742 (2.419-3.032) | 67 (55-81) | 2.956 (2.415-3.595) | -0.43 (-0.91 to 0.06) |
| Kyrgyzstan | 48 (40-56) | 1.716 (1.435-2.021) | 61 (49-75) | 1.372 (1.103-1.688) | -1.48 (-1.86 to -1.09) |
| Lao People's Democratic Republic | 28 (20-41) | 1.591 (1.115-2.233) | 50 (35-75) | 1.296 (0.918-1.973) | -0.74 (-0.85 to -0.64) |
| Latvia | 140 (130-150) | 3.9 (3.637-4.174) | 203 (176-234) | 4.638 (4.008-5.366) | 0.54 (0.33 to 0.74) |
| Lebanon | 182 (127-247) | 9.913 (7.021-13.414) | 511 (391-649) | 7.832 (6.042-9.95) | -0.36 (-0.58 to -0.14) |
| Lesotho | 13 (10-19) | 1.75 (1.331-2.425) | 23 (14-32) | 2.336 (1.53-3.249) | 1.41 (1.15 to 1.68) |
| Liberia | 25 (18-33) | 2.51 (1.892-3.294) | 35 (25-49) | 2.07 (1.531-2.863) | -0.92 (-1.05 to -0.79) |
| Libya | 93 (65-131) | 5.697 (3.89-7.908) | 270 (195-374) | 6.289 (4.491-8.657) | 0.66 (0.48 to 0.84) |
| Lithuania | 195 (182-207) | 4.318 (4.03-4.593) | 266 (231-297) | 4.125 (3.58-4.634) | -0.83 (-1.55 to -0.11) |
| Luxembourg | 33 (31-35) | 5.923 (5.539-6.248) | 45 (40-50) | 3.872 (3.446-4.317) | -1.17 (-1.3 to -1.03) |
| Madagascar | 102 (85-123) | 2.403 (2.02-2.913) | 168 (123-213) | 2.005 (1.485-2.539) | -0.63 (-0.74 to -0.53) |
| Malawi | 236 (192-282) | 7.638 (6.356-9.133) | 521 (407-657) | 8.604 (6.873-10.688) | 0.32 (0.16 to 0.47) |
| Malaysia | 204 (130-269) | 2.507 (1.587-3.341) | 651 (473-804) | 2.66 (1.906-3.293) | -0.06 (-0.2 to 0.08) |
| Maldives | 1 (1-1) | 1.792 (1.355-2.381) | 3 (2-4) | 1.094 (0.843-1.375) | -1.88 (-1.98 to -1.78) |
| Mali | 285 (233-345) | 9.15 (7.593-10.848) | 619 (469-815) | 8.994 (6.956-11.522) | 0.07 (0.01 to 0.14) |
| Malta | 27 (24-29) | 6.376 (5.814-6.878) | 41 (35-46) | 3.763 (3.249-4.254) | -1.73 (-1.87 to -1.59) |
| Marshall Islands | 0 (0-0) | 1.6 (1.211-2.065) | 1 (0-1) | 2.034 (1.406-2.728) | 0.98 (0.85 to 1.12) |
| Mauritania | 21 (16-27) | 2.418 (1.914-3.113) | 36 (24-52) | 1.992 (1.347-2.831) | -0.98 (-1.12 to -0.84) |
| Mauritius | 31 (29-33) | 5.011 (4.697-5.3) | 39 (36-42) | 2.24 (2.086-2.386) | -1.53 (-2.32 to -0.73) |
| Mexico | 621 (601-637) | 1.719 (1.647-1.77) | 1641 (1446-1849) | 1.388 (1.224-1.561) | -0.57 (-0.73 to -0.42) |
| Micronesia (Federated States of) | 1 (1-1) | 1.72 (1.266-2.212) | 1 (1-2) | 1.921 (1.339-2.569) | 0.4 (0.36 to 0.44) |
| Monaco | 4 (3-6) | 5.195 (3.445-7.189) | 6 (3-14) | 5.605 (2.65-12.354) | 0.34 (0.31 to 0.38) |
| Mongolia | 23 (15-31) | 2.274 (1.469-3.028) | 25 (19-32) | 1.207 (0.918-1.566) | -2.87 (-3.34 to -2.4) |
| Montenegro | 22 (17-28) | 3.685 (2.895-4.634) | 44 (34-56) | 4.662 (3.649-5.904) | 0.81 (0.62 to 1) |
| Morocco | 164 (113-212) | 1.307 (0.888-1.688) | 435 (308-571) | 1.456 (1.032-1.9) | 0.42 (0.28 to 0.56) |
| Mozambique | 133 (105-172) | 2.807 (2.219-3.579) | 270 (199-362) | 3.071 (2.327-4.029) | 0.6 (0.49 to 0.7) |
| Myanmar | 306 (229-400) | 1.537 (1.19-1.997) | 494 (357-777) | 1.154 (0.842-1.858) | -1.1 (-1.2 to -1) |
| Namibia | 8 (7-10) | 1.565 (1.273-1.89) | 20 (15-25) | 1.698 (1.349-2.128) | 0.11 (-0.11 to 0.33) |
| Nauru | 0 (0-0) | 2.372 (1.701-3.151) | 0 (0-0) | 2.346 (1.567-3.184) | 0 (-0.1 to 0.1) |
| Nepal | 96 (63-147) | 1.301 (0.851-1.952) | 244 (163-483) | 1.237 (0.836-2.422) | -0.15 (-0.39 to 0.09) |
| Netherlands | 1199 (1110-1278) | 5.782 (5.357-6.151) | 1642 (1440-1807) | 4.194 (3.7-4.6) | -0.97 (-1.11 to -0.83) |
| New Zealand | 162 (147-176) | 4.082 (3.702-4.427) | 287 (252-316) | 3.126 (2.752-3.446) | -1.13 (-1.43 to -0.84) |
| Nicaragua | 8 (7-10) | 0.612 (0.521-0.717) | 27 (22-33) | 0.608 (0.495-0.739) | 0.22 (-0.03 to 0.48) |
| Niger | 38 (28-57) | 1.779 (1.332-2.631) | 91 (64-144) | 1.455 (1.038-2.257) | -0.83 (-0.93 to -0.73) |
| Nigeria | 259 (204-365) | 0.706 (0.562-0.994) | 528 (424-705) | 0.755 (0.609-1.048) | 0.29 (0.24 to 0.34) |
| Niue | 0 (0-0) | 1.437 (1.091-1.766) | 0 (0-0) | 1.768 (1.28-2.215) | 0.65 (0.57 to 0.73) |
| North Macedonia | 81 (68-99) | 4.752 (4.017-5.774) | 155 (118-197) | 5.139 (4.002-6.383) | 0.14 (-0.19 to 0.47) |
| Northern Mariana Islands | 0 (0-0) | 1.4 (1.08-2.3) | 1 (1-2) | 3.279 (2.494-3.76) | 4 (3.38 to 4.63) |
| Norway | 418 (389-437) | 5.552 (5.196-5.802) | 431 (379-466) | 3.742 (3.33-4.037) | -1.32 (-1.44 to -1.2) |
| Oman | 10 (7-13) | 1.794 (1.277-2.44) | 21 (16-26) | 1.494 (1.132-1.925) | -0.2 (-0.42 to 0.01) |
| Pakistan | 2223 (1750-2717) | 4.555 (3.572-5.602) | 5026 (3939-6639) | 5.169 (4.036-6.9) | 0.18 (0.02 to 0.35) |
| Palau | 0 (0-0) | 0.615 (0.473-0.768) | 0 (0-0) | 0.576 (0.438-0.733) | -0.16 (-0.24 to -0.08) |
| Palestine | 31 (23-41) | 4.15 (3.088-5.438) | 66 (52-83) | 3.256 (2.6-4.158) | -0.93 (-1.17 to -0.69) |
| Panama | 16 (14-17) | 1.134 (1.038-1.236) | 51 (40-61) | 1.137 (0.891-1.364) | -0.11 (-0.32 to 0.1) |
| Papua New Guinea | 17 (10-25) | 1.135 (0.683-1.651) | 54 (32-83) | 1.291 (0.778-1.958) | 0.51 (0.47 to 0.56) |
| Paraguay | 23 (18-28) | 1.125 (0.902-1.409) | 79 (58-103) | 1.465 (1.076-1.915) | 1.14 (1.04 to 1.23) |
| Peru | 174 (142-215) | 1.601 (1.307-1.973) | 444 (331-591) | 1.347 (1.005-1.794) | -0.75 (-0.97 to -0.52) |
| Philippines | 218 (183-265) | 0.909 (0.754-1.108) | 667 (541-922) | 0.907 (0.737-1.287) | 0.21 (0.16 to 0.26) |
| Poland | 2231 (2146-2314) | 5.116 (4.909-5.316) | 5219 (4705-5711) | 6.814 (6.155-7.456) | 0.63 (0.48 to 0.78) |
| Portugal | 692 (647-735) | 5.194 (4.853-5.52) | 1186 (1045-1293) | 4.105 (3.675-4.458) | -0.72 (-0.84 to -0.6) |
| Puerto Rico | 103 (94-110) | 2.954 (2.705-3.158) | 177 (145-208) | 2.158 (1.769-2.532) | -0.85 (-1.03 to -0.66) |
| Qatar | 4 (3-5) | 5.513 (4.272-7.1) | 17 (12-25) | 3.281 (2.337-4.678) | -1.85 (-2.36 to -1.34) |
| Republic of Côte d'Ivoire | 76 (58-92) | 2.622 (2.073-3.108) | 210 (158-293) | 2.455 (1.936-3.326) | -0.32 (-0.44 to -0.21) |
| Republic of Korea | 713 (577-823) | 3.08 (2.524-3.527) | 1968 (1468-2415) | 2.113 (1.575-2.591) | -1.49 (-1.63 to -1.36) |
| Republic of Moldova | 138 (128-149) | 3.41 (3.159-3.711) | 186 (167-207) | 3.043 (2.726-3.392) | -0.62 (-0.84 to -0.39) |
| Republic of South Sudan | 58 (43-81) | 2.606 (1.964-3.573) | 77 (55-106) | 2.57 (1.845-3.478) | -0.18 (-0.29 to -0.07) |
| Romania | 1010 (937-1076) | 3.72 (3.46-3.967) | 1910 (1685-2163) | 4.824 (4.267-5.443) | 0.74 (0.62 to 0.86) |
| Russian Federation | 5579 (5399-5724) | 3.13 (3.025-3.214) | 6904 (6284-7555) | 2.827 (2.573-3.087) | -0.74 (-1.07 to -0.41) |
| Rwanda | 80 (62-98) | 3.439 (2.712-4.247) | 123 (83-187) | 2.493 (1.691-3.7) | -1.68 (-1.91 to -1.45) |
| Saint Kitts and Nevis | 1 (1-1) | 3.613 (3.334-3.839) | 2 (1-2) | 3.169 (2.713-3.54) | 0.27 (0.05 to 0.49) |
| Saint Lucia | 3 (3-4) | 4.284 (4.019-4.554) | 7 (6-9) | 3.165 (2.613-3.734) | -1.11 (-1.39 to -0.84) |
| Saint Vincent and the Grenadines | 2 (2-2) | 2.659 (2.452-2.864) | 4 (3-4) | 2.642 (2.321-2.957) | 0.38 (0.12 to 0.63) |
| Samoa | 1 (1-1) | 1.264 (1.008-1.698) | 2 (1-2) | 1.213 (0.883-1.616) | -0.22 (-0.25 to -0.18) |
| San Marino | 3 (2-4) | 7.728 (6.121-9.392) | 4 (2-5) | 3.797 (2.536-5.337) | -1.18 (-1.6 to -0.77) |
| Sao Tome and Principe | 2 (2-2) | 3.496 (2.94-4.13) | 4 (3-5) | 4.483 (3.321-5.866) | 0.98 (0.87 to 1.09) |
| Saudi Arabia | 94 (67-132) | 2.009 (1.457-2.741) | 226 (156-392) | 1.704 (1.219-2.71) | -0.84 (-1 to -0.69) |
| Senegal | 71 (56-88) | 2.601 (2.076-3.194) | 148 (111-200) | 2.276 (1.708-3.062) | -0.67 (-0.76 to -0.57) |
| Serbia | 533 (426-655) | 5.728 (4.584-7.027) | 858 (658-1084) | 4.911 (3.774-6.182) | -0.42 (-0.49 to -0.36) |
| Seychelles | 3 (2-3) | 4.876 (4.211-5.565) | 4 (3-5) | 3.846 (3.217-4.787) | -0.3 (-0.52 to -0.07) |
| Sierra Leone | 42 (32-53) | 2.32 (1.791-2.892) | 61 (46-82) | 1.909 (1.47-2.526) | -0.8 (-0.88 to -0.71) |
| Singapore | 42 (39-45) | 2.226 (2.051-2.397) | 116 (101-129) | 1.42 (1.223-1.573) | -1.54 (-1.78 to -1.3) |
| Slovakia | 289 (243-346) | 4.824 (4.073-5.74) | 394 (310-508) | 4.032 (3.176-5.163) | -0.36 (-0.43 to -0.28) |
| Slovenia | 104 (95-112) | 4.199 (3.843-4.512) | 224 (191-254) | 4.461 (3.832-5.083) | -0.04 (-0.21 to 0.12) |
| Solomon Islands | 2 (1-2) | 1.498 (0.847-2.206) | 5 (3-7) | 1.688 (1.051-2.396) | 0.46 (0.41 to 0.5) |
| Somalia | 51 (37-70) | 2.831 (2.085-3.9) | 120 (85-159) | 2.59 (1.866-3.399) | -0.24 (-0.29 to -0.2) |
| South Africa | 388 (304-507) | 2.048 (1.585-2.703) | 948 (821-1071) | 2.302 (1.972-2.584) | 0.31 (0.09 to 0.53) |
| Spain | 3965 (3682-4225) | 7.086 (6.597-7.546) | 5851 (5115-6491) | 5.036 (4.48-5.538) | -0.98 (-1.11 to -0.86) |
| Sri Lanka | 100 (81-124) | 1.152 (0.942-1.42) | 238 (156-342) | 0.951 (0.631-1.343) | -0.19 (-0.39 to 0.02) |
| Sudan | 245 (167-381) | 3.05 (2.072-4.679) | 439 (331-586) | 2.693 (2.071-3.55) | -0.48 (-0.55 to -0.4) |
| Suriname | 4 (3-5) | 1.687 (1.376-2.056) | 10 (7-13) | 1.59 (1.152-2.17) | 0.22 (0.04 to 0.4) |
| Sweden | 661 (607-705) | 3.952 (3.634-4.197) | 883 (761-990) | 3.375 (2.932-3.779) | -0.15 (-0.36 to 0.05) |
| Switzerland | 301 (272-330) | 2.689 (2.451-2.935) | 651 (553-735) | 3.028 (2.616-3.414) | 0.25 (-0.04 to 0.53) |
| Syrian Arab Republic | 104 (79-139) | 2.325 (1.743-3.116) | 259 (181-355) | 2.47 (1.759-3.348) | 0 (-0.12 to 0.13) |
| Taiwan (Province of China) | 521 (481-553) | 3.849 (3.509-4.111) | 1398 (1218-1551) | 3.2 (2.8-3.554) | -1.06 (-1.31 to -0.8) |
| Tajikistan | 24 (17-37) | 0.959 (0.655-1.5) | 36 (26-48) | 0.742 (0.551-0.972) | -0.96 (-1.24 to -0.67) |
| Thailand | 787 (629-960) | 2.695 (2.154-3.28) | 2223 (1664-2885) | 2.051 (1.54-2.662) | -1.38 (-1.53 to -1.22) |
| Timor-Leste | 2 (2-3) | 1.044 (0.764-1.391) | 8 (5-11) | 1.013 (0.726-1.559) | -0.02 (-0.19 to 0.16) |
| Togo | 24 (19-29) | 2.445 (1.994-2.916) | 64 (48-85) | 2.108 (1.629-2.755) | -0.79 (-0.94 to -0.64) |
| Tokelau | 0 (0-0) | 1.563 (1.152-1.993) | 0 (0-0) | 1.582 (1.104-2.135) | 0.09 (0.02 to 0.16) |
| Tonga | 1 (0-1) | 1.225 (0.802-1.869) | 1 (1-2) | 1.44 (0.918-2.186) | 0.57 (0.42 to 0.73) |
| Trinidad and Tobago | 17 (16-18) | 2.28 (2.157-2.4) | 35 (27-44) | 1.843 (1.43-2.315) | -0.36 (-0.54 to -0.19) |
| Tunisia | 153 (114-199) | 3.682 (2.729-4.729) | 422 (287-588) | 3.472 (2.377-4.783) | -0.44 (-0.55 to -0.33) |
| Turkey | 1504 (1171-1913) | 4.94 (3.88-6.232) | 3514 (2730-4405) | 3.984 (3.122-4.976) | -0.82 (-1.07 to -0.56) |
| Turkmenistan | 27 (24-31) | 1.527 (1.346-1.722) | 43 (33-56) | 1.156 (0.9-1.484) | -1.58 (-1.94 to -1.22) |
| Tuvalu | 0 (0-0) | 1.475 (1.096-1.866) | 0 (0-0) | 1.704 (1.191-2.17) | 0.61 (0.53 to 0.69) |
| Uganda | 162 (125-204) | 3.042 (2.365-3.79) | 352 (266-457) | 2.992 (2.306-3.809) | -0.43 (-0.59 to -0.27) |
| Ukraine | 1942 (1636-2416) | 2.69 (2.274-3.323) | 2253 (1631-3001) | 2.81 (2.029-3.739) | -0.09 (-0.35 to 0.17) |
| United Arab Emirates | 19 (11-32) | 5.459 (3.239-9.192) | 91 (68-121) | 4.601 (3.523-5.816) | 0.94 (0.39 to 1.48) |
| United Kingdom | 6406 (6096-6575) | 6.65 (6.331-6.825) | 6885 (6120-7277) | 4.57 (4.113-4.81) | -1.18 (-1.33 to -1.04) |
| United Republic of Tanzania | 258 (204-340) | 2.866 (2.283-3.725) | 508 (377-687) | 2.388 (1.833-3.18) | -0.79 (-0.86 to -0.72) |
| United States of America | 11814 (10912-12350) | 3.519 (3.257-3.677) | 21497 (19023-22907) | 3.413 (3.039-3.623) | -0.03 (-0.11 to 0.06) |
| United States Virgin Islands | 1 (1-2) | 1.683 (1.33-2.093) | 2 (2-3) | 1.172 (0.869-1.502) | -1.08 (-1.3 to -0.85) |
| Uruguay | 232 (216-248) | 5.839 (5.438-6.237) | 312 (282-340) | 5.05 (4.598-5.509) | -0.65 (-0.77 to -0.54) |
| Uzbekistan | 115 (78-162) | 1.036 (0.696-1.489) | 276 (214-354) | 1.176 (0.92-1.497) | 0.39 (0.2 to 0.58) |
| Vanuatu | 1 (0-1) | 1.458 (0.961-2.091) | 2 (2-3) | 1.631 (1.115-2.208) | 0.37 (0.33 to 0.41) |
| Venezuela (Bolivarian Republic of) | 156 (146-164) | 1.808 (1.68-1.901) | 516 (400-656) | 1.836 (1.43-2.328) | -0.21 (-0.34 to -0.09) |
| Viet Nam | 381 (289-478) | 1.03 (0.786-1.294) | 1038 (810-1249) | 1.182 (0.928-1.425) | 0.4 (0.35 to 0.46) |
| Yemen | 119 (81-171) | 2.901 (1.966-4.129) | 339 (242-456) | 2.948 (2.105-3.983) | 0 (-0.05 to 0.04) |
| Zambia | 71 (58-88) | 3.051 (2.481-3.792) | 198 (120-344) | 3.5 (2.265-5.688) | 0.38 (0.26 to 0.49) |
| Zimbabwe | 221 (177-265) | 6.714 (5.408-7.997) | 440 (318-574) | 7.838 (5.742-9.919) | 0.79 (0.5 to 1.08) |
| Disability-adjusted life years |  |  |  |  |  |
| Afghanistan | 6148 (3558-9081) | 89.825 (54.475-129.373) | 7608 (4438-10734) | 77.677 (47.645-105.354) | -0.58 (-0.64 to -0.53) |
| Albania | 129 (104-162) | 6.606 (5.313-8.305) | 256 (192-348) | 5.754 (4.342-7.804) | -0.16 (-0.31 to -0.01) |
| Algeria | 3791 (2804-4731) | 36.943 (26.642-45.771) | 9511 (7294-12316) | 30.511 (23.612-38.985) | -0.44 (-0.59 to -0.28) |
| American Samoa | 7 (6-9) | 29.057 (24.132-38.473) | 21 (18-26) | 44.001 (36.621-53.161) | 2.17 (1.77 to 2.56) |
| Andorra | 70 (49-101) | 121.94 (84.816-175.55) | 117 (78-161) | 74.555 (49.574-103.27) | -1.38 (-1.63 to -1.13) |
| Angola | 2162 (1578-2949) | 57.743 (43.681-78.119) | 5966 (4400-7926) | 52.736 (39.46-69.637) | -0.28 (-0.38 to -0.18) |
| Antigua and Barbuda | 29 (27-31) | 53.23 (49.392-56.993) | 54 (50-58) | 52.288 (47.877-55.685) | 0.23 (-0.02 to 0.47) |
| Argentina | 32249 (30288-34201) | 99.724 (93.666-105.475) | 34111 (31292-36868) | 60.423 (55.452-65.234) | -1.42 (-1.53 to -1.31) |
| Armenia | 3034 (2595-3599) | 109.923 (93.943-130.802) | 4882 (4014-5770) | 112.347 (92.472-132.8) | -0.03 (-0.28 to 0.22) |
| Australia | 15319 (14377-16258) | 77.715 (73.029-82.377) | 22833 (20498-25071) | 48.199 (43.679-52.691) | -1.51 (-1.59 to -1.44) |
| Austria | 11603 (10804-12290) | 96.254 (90.13-101.89) | 11740 (10562-12808) | 62.088 (56.438-67.526) | -1.22 (-1.32 to -1.11) |
| Azerbaijan | 2605 (1961-3552) | 49.904 (38.154-67.619) | 4281 (2964-5990) | 40.646 (28.58-55.457) | -0.7 (-0.84 to -0.56) |
| Bahamas | 56 (51-61) | 36.241 (33.385-39.4) | 150 (122-186) | 37.577 (30.592-46.234) | 0.47 (0.33 to 0.6) |
| Bahrain | 250 (200-307) | 159.57 (128.132-196.691) | 748 (541-1075) | 96.7 (70.045-132.259) | -2.15 (-2.39 to -1.9) |
| Bangladesh | 14197 (10078-21198) | 31.117 (21.891-46.736) | 29299 (17695-54898) | 22.037 (13.452-41.711) | -1.3 (-1.46 to -1.14) |
| Barbados | 144 (133-155) | 48.174 (44.785-51.364) | 254 (202-311) | 48.587 (38.6-59.616) | 0.36 (0.21 to 0.51) |
| Belarus | 9756 (8961-10548) | 74.458 (68.604-80.382) | 9100 (7345-10921) | 55.719 (45.184-67.139) | -1.74 (-2.14 to -1.33) |
| Belgium | 19995 (18602-21176) | 128.186 (119.953-135.641) | 19238 (17108-21083) | 79.485 (71.839-86.471) | -1.5 (-1.72 to -1.28) |
| Belize | 31 (29-33) | 33.506 (31.276-35.81) | 120 (105-137) | 40.836 (35.462-46.329) | 0.85 (0.45 to 1.26) |
| Benin | 956 (749-1203) | 48.648 (38.368-61.145) | 1923 (1462-2537) | 37.272 (28.945-48.659) | -1.11 (-1.25 to -0.97) |
| Bermuda | 82 (76-89) | 134.076 (124.276-144.4) | 121 (103-145) | 86.527 (73.021-103.774) | -0.94 (-1.21 to -0.67) |
| Bhutan | 59 (37-93) | 25.544 (16.113-40.851) | 131 (85-267) | 22.29 (14.473-45.692) | -0.53 (-0.63 to -0.43) |
| Bolivia (Plurinational State of) | 1544 (1099-2028) | 49.997 (35.666-65.563) | 3796 (2756-5253) | 43.359 (31.584-59.853) | -0.45 (-0.51 to -0.4) |
| Bosnia and Herzegovina | 2787 (2383-3284) | 68.442 (58.925-80.013) | 5466 (4047-6989) | 85.464 (63.25-109.704) | 0.97 (0.8 to 1.14) |
| Botswana | 304 (227-393) | 54.427 (41.11-68.493) | 648 (476-903) | 43.925 (33.746-59.059) | -0.88 (-1.08 to -0.68) |
| Brazil | 46371 (44388-48343) | 53.775 (51.186-56.117) | 118785 (110164-124955) | 47.644 (44.071-50.155) | -0.35 (-0.41 to -0.29) |
| Brunei Darussalam | 62 (50-77) | 64.636 (52.27-79.472) | 142 (115-174) | 45.955 (38.002-56.39) | -0.51 (-0.73 to -0.29) |
| Bulgaria | 10569 (9335-11810) | 85.686 (76.772-94.896) | 15914 (13494-18926) | 111.314 (93.978-133.065) | 1.12 (1.01 to 1.23) |
| Burkina Faso | 1999 (1448-2663) | 48.337 (35.615-63.699) | 3546 (2689-4910) | 38.625 (29.365-53.436) | -0.95 (-1.09 to -0.8) |
| Burundi | 1633 (1221-2081) | 70.77 (53.605-90.321) | 2345 (1718-3209) | 50.035 (37.234-67.901) | -1.51 (-1.67 to -1.36) |
| Cabo Verde | 12 (9-30) | 5.394 (3.667-13.013) | 191 (96-257) | 42.547 (21.87-56.757) | 5.51 (4.05 to 6.99) |
| Cambodia | 1460 (1124-1931) | 32.49 (25.314-42.785) | 3621 (2599-5095) | 29.579 (21.222-42.14) | -0.39 (-0.58 to -0.2) |
| Cameroon | 2379 (1904-2888) | 56.141 (45.833-67.62) | 6400 (4647-8686) | 50.203 (38.28-68.373) | -0.61 (-0.76 to -0.46) |
| Canada | 34116 (31497-36257) | 104.452 (96.466-111.049) | 45285 (40821-49439) | 60.272 (54.522-65.641) | -1.73 (-1.88 to -1.58) |
| Central African Republic | 777 (542-1050) | 69.361 (52.008-90.596) | 1317 (900-1808) | 59.741 (44.176-78.196) | -0.53 (-0.58 to -0.49) |
| Chad | 1144 (866-1449) | 41.218 (31.208-52.119) | 2607 (1912-3448) | 45.481 (33.588-59.331) | 0.07 (-0.12 to 0.26) |
| Chile | 6368 (5996-6742) | 64.495 (60.741-68.346) | 12207 (11212-13103) | 47.053 (43.238-50.517) | -0.77 (-0.89 to -0.65) |
| China | 561800 (400978-659992) | 69.946 (50.985-81.444) | 930100 (735478-1185431) | 45.313 (36.058-57.411) | -1.74 (-1.88 to -1.6) |
| Colombia | 7099 (6676-7525) | 41.059 (38.603-43.476) | 15633 (13010-18620) | 28.416 (23.661-33.865) | -1.54 (-1.72 to -1.35) |
| Comoros | 98 (70-134) | 51.106 (36.912-69.242) | 217 (149-316) | 45.867 (31.504-66.84) | -0.58 (-0.71 to -0.46) |
| Congo | 770 (516-1070) | 74.682 (53.739-101.302) | 1648 (1195-2222) | 63.362 (47.392-83.183) | -0.71 (-0.87 to -0.55) |
| Cook Islands | 9 (7-11) | 72.085 (59.324-85) | 17 (13-21) | 66.147 (52.393-82.974) | -0.09 (-0.22 to 0.03) |
| Costa Rica | 781 (721-837) | 45.512 (41.938-48.77) | 2146 (1870-2414) | 39.112 (34.109-43.921) | -0.79 (-0.93 to -0.64) |
| Croatia | 6170 (5551-6777) | 104.161 (93.795-114.227) | 9839 (8357-11497) | 106.955 (91.195-124.528) | 0.26 (0.12 to 0.4) |
| Cuba | 7520 (7015-7950) | 73.589 (68.854-77.706) | 15726 (13421-17832) | 79.23 (67.802-89.947) | 0.53 (0.37 to 0.69) |
| Cyprus | 861 (703-1082) | 123.201 (99.869-155.005) | 1610 (1250-2063) | 77.185 (60.519-98.446) | -1.38 (-1.46 to -1.3) |
| Czechia | 15875 (14179-17824) | 113.702 (101.862-127.364) | 21542 (17997-25496) | 97.061 (80.867-115.621) | -0.58 (-0.67 to -0.48) |
| Democratic People's Republic of Korea | 7350 (5361-9763) | 45.749 (33.748-60.124) | 13494 (10089-19859) | 40.829 (30.486-59.888) | -0.29 (-0.35 to -0.24) |
| Democratic Republic of the Congo | 8428 (6592-10791) | 57.764 (44.338-73.398) | 19167 (13913-25451) | 54.127 (39.447-72.255) | -0.23 (-0.42 to -0.04) |
| Denmark | 8843 (7632-10589) | 110.036 (95.537-130.493) | 10801 (9817-11799) | 87.487 (79.878-95.141) | -1.67 (-2.21 to -1.13) |
| Djibouti | 71 (51-98) | 55.051 (40.325-74.526) | 335 (222-503) | 56.036 (38.308-81.609) | -0.06 (-0.14 to 0.03) |
| Dominica | 41 (34-48) | 68.066 (56.452-80.28) | 57 (45-70) | 68.351 (54.726-83.964) | 0.18 (0.09 to 0.26) |
| Dominican Republic | 760 (616-898) | 21.501 (17.427-25.384) | 2308 (1682-3153) | 23.252 (16.966-31.721) | 0.61 (0.48 to 0.74) |
| Ecuador | 1304 (1206-1420) | 25.38 (23.441-27.683) | 4574 (3585-5855) | 28.441 (22.382-36.28) | 0.38 (0.11 to 0.64) |
| Egypt | 56912 (33666-67654) | 194.814 (131.97-229.763) | 55029 (39999-90226) | 91.715 (65.829-153.702) | -3.06 (-3.4 to -2.73) |
| El Salvador | 546 (477-619) | 18.348 (15.954-20.833) | 1247 (997-1531) | 20.103 (16.031-24.697) | 0.25 (0.14 to 0.36) |
| Equatorial Guinea | 122 (86-163) | 64.561 (47.664-84.973) | 284 (193-405) | 57.92 (40.37-80.636) | -0.32 (-0.56 to -0.09) |
| Eritrea | 724 (489-973) | 64.471 (45.343-85.298) | 1634 (1185-2237) | 60.815 (45.481-81.097) | -0.29 (-0.36 to -0.23) |
| Estonia | 1777 (1627-1940) | 86.488 (79.252-94.369) | 2037 (1754-2349) | 73.316 (63.318-84.187) | -1.1 (-1.35 to -0.84) |
| Eswatini | 172 (133-226) | 59.232 (45.7-76.828) | 379 (252-534) | 63.615 (43.471-87.258) | 0.49 (0.09 to 0.9) |
| Ethiopia | 13038 (10252-17625) | 67.806 (52.735-91.267) | 20174 (14193-28920) | 48.414 (34.063-68.923) | -1.39 (-1.54 to -1.24) |
| Fiji | 117 (85-144) | 31.224 (22.796-38.408) | 301 (184-406) | 38.455 (23.758-51.123) | 1.04 (0.84 to 1.25) |
| Finland | 5585 (5200-5954) | 77.353 (72.196-82.331) | 5812 (5250-6387) | 43.769 (39.945-47.895) | -2.07 (-2.18 to -1.96) |
| France | 104940 (98462-111152) | 126.948 (119.34-134.32) | 129124 (114123-144599) | 89.007 (79.528-99.784) | -1.05 (-1.11 to -1) |
| Gabon | 434 (336-560) | 77.779 (60.696-99.856) | 704 (517-948) | 69.395 (51.734-93.198) | -0.46 (-0.51 to -0.41) |
| Gambia | 95 (69-121) | 28.001 (21.004-35.509) | 282 (202-367) | 29.617 (21.374-38.312) | 0.03 (-0.09 to 0.16) |
| Georgia | 5819 (4752-6951) | 91.976 (75.24-109.76) | 6787 (5824-7754) | 115.521 (98.94-132.029) | 0.73 (0.18 to 1.3) |
| Germany | 137099 (127802-145176) | 105.865 (98.877-111.884) | 136409 (122859-148047) | 67.877 (62.048-73.351) | -1.57 (-1.76 to -1.37) |
| Ghana | 3518 (2505-4628) | 55.278 (41.723-70.656) | 8819 (6629-11609) | 50.214 (37.486-66.157) | -0.41 (-0.53 to -0.29) |
| Greece | 23271 (21743-24556) | 150.245 (140.151-158.373) | 28801 (26297-30972) | 116.045 (106.998-124.779) | -0.92 (-1.1 to -0.75) |
| Greenland | 35 (24-42) | 112.444 (77.077-132.99) | 48 (33-62) | 70.954 (49.769-91.108) | -1.27 (-1.42 to -1.13) |
| Grenada | 39 (35-43) | 53.813 (48.312-59.838) | 64 (54-73) | 56.663 (47.744-64.018) | 0.45 (0.17 to 0.74) |
| Guam | 24 (20-29) | 29.883 (25.247-36.926) | 66 (56-78) | 32.253 (27-37.837) | 1.37 (1.01 to 1.73) |
| Guatemala | 710 (679-740) | 21.688 (20.783-22.629) | 1741 (1475-2011) | 15.866 (13.498-18.267) | -1.16 (-1.4 to -0.92) |
| Guinea | 1970 (1498-2441) | 61.371 (46.823-75.904) | 3810 (2804-5209) | 70.012 (52.283-95.397) | 0.55 (0.48 to 0.63) |
| Guinea-Bissau | 269 (173-366) | 68.566 (45.932-91.953) | 423 (303-562) | 55.887 (41.38-72.961) | -0.8 (-0.88 to -0.72) |
| Guyana | 134 (118-150) | 36.159 (32.088-40.431) | 234 (174-306) | 36.428 (27.519-46.871) | 0.6 (0.41 to 0.8) |
| Haiti | 2020 (1344-2737) | 65.045 (45.235-87.166) | 4099 (2807-5694) | 60.143 (42.473-81.328) | -0.08 (-0.15 to 0) |
| Honduras | 219 (175-269) | 10.674 (8.529-13.112) | 898 (676-1193) | 14.529 (10.867-19.316) | 1.2 (1.08 to 1.32) |
| Hungary | 17277 (15613-18876) | 116.619 (105.526-127.662) | 22706 (19743-26172) | 117.438 (102.287-135.014) | -0.05 (-0.3 to 0.2) |
| Iceland | 283 (259-306) | 97.662 (89.79-105.455) | 364 (318-409) | 60.439 (53.37-68.061) | -1.5 (-1.64 to -1.35) |
| India | 115854 (89299-137025) | 25.844 (20.088-30.708) | 289346 (250585-342397) | 24.937 (21.659-29.367) | -0.37 (-0.53 to -0.21) |
| Indonesia | 23945 (19418-30215) | 24.451 (19.484-32.024) | 57247 (38611-92345) | 24.596 (16.686-40.599) | -0.03 (-0.05 to -0.01) |
| Iran (Islamic Republic of) | 12372 (10091-15003) | 49.432 (40.294-59.954) | 34242 (28274-38987) | 45.6 (37.595-52.038) | -0.1 (-0.18 to -0.02) |
| Iraq | 7563 (5630-9945) | 96.306 (71.849-126.697) | 24712 (17557-33117) | 110.076 (79.353-146.451) | 0.22 (0.1 to 0.35) |
| Ireland | 3624 (3383-3854) | 87.406 (81.557-92.728) | 4561 (4005-5084) | 55.664 (49.017-61.899) | -1.32 (-1.48 to -1.15) |
| Israel | 4464 (4115-4802) | 91.941 (84.86-98.712) | 8841 (7819-9701) | 69.571 (61.987-76.138) | -0.99 (-1.26 to -0.72) |
| Italy | 135126 (128640-141292) | 149.067 (141.959-155.765) | 144570 (129784-156762) | 92.637 (84.877-100.095) | -1.55 (-1.61 to -1.49) |
| Jamaica | 869 (799-940) | 47.775 (44.064-51.541) | 1590 (1207-2050) | 51.243 (38.84-66.22) | 0.53 (0.21 to 0.84) |
| Japan | 76940 (72497-80413) | 46.028 (43.098-48.149) | 166167 (143649-180400) | 40.574 (36.57-43.406) | -0.33 (-0.39 to -0.26) |
| Jordan | 889 (697-1120) | 69.66 (54.977-87.591) | 3783 (2688-5278) | 53.027 (38.313-72.537) | -1.06 (-1.2 to -0.91) |
| Kazakhstan | 7650 (5913-10154) | 59.039 (45.732-78.217) | 8233 (6908-9724) | 45.128 (37.887-53.356) | -1.59 (-2.11 to -1.07) |
| Kenya | 1534 (1134-1941) | 19.348 (14.303-24.429) | 5537 (4375-6748) | 25.306 (20.432-30.428) | 1.1 (0.94 to 1.25) |
| Kiribati | 5 (4-6) | 12.012 (9.401-14.467) | 10 (7-13) | 13.403 (9.739-16.818) | 0.41 (0.33 to 0.48) |
| Kuwait | 317 (284-350) | 56.678 (50.149-62.68) | 1669 (1376-2062) | 59.47 (48.745-72.941) | -0.65 (-1.16 to -0.13) |
| Kyrgyzstan | 1194 (982-1390) | 40.107 (33.065-46.827) | 1641 (1313-2035) | 32.849 (26.327-40.45) | -1.55 (-2.01 to -1.1) |
| Lao People's Democratic Republic | 742 (510-1076) | 35.63 (24.905-51.307) | 1249 (872-1813) | 27.332 (19.155-40.654) | -0.95 (-1.05 to -0.84) |
| Latvia | 3090 (2873-3313) | 86.218 (80.065-92.471) | 3911 (3350-4536) | 98.334 (83.693-114.57) | 0.25 (0.02 to 0.47) |
| Lebanon | 4068 (2767-5549) | 197.27 (137.293-268.099) | 9150 (7201-11525) | 147.627 (116.798-186.45) | -0.56 (-0.76 to -0.36) |
| Lesotho | 315 (239-441) | 37.501 (28.573-52.486) | 602 (376-865) | 54.1 (34.509-76.281) | 1.65 (1.35 to 1.94) |
| Liberia | 555 (403-765) | 49.469 (36.501-66.933) | 906 (633-1351) | 41.93 (30.216-59.186) | -0.81 (-0.95 to -0.68) |
| Libya | 2060 (1458-2873) | 113.957 (80.443-158.738) | 6320 (4534-8608) | 127.333 (91.693-174.486) | 0.59 (0.42 to 0.76) |
| Lithuania | 4170 (3876-4451) | 92.476 (86.197-98.516) | 4882 (4226-5489) | 83.556 (72.32-93.999) | -1.05 (-1.71 to -0.39) |
| Luxembourg | 652 (613-692) | 118.478 (111.683-125.426) | 787 (706-879) | 71.974 (64.303-80.559) | -1.42 (-1.54 to -1.3) |
| Madagascar | 2510 (2087-2997) | 50.553 (42.339-60.894) | 4517 (3270-5797) | 41.362 (30.358-52.332) | -0.7 (-0.81 to -0.6) |
| Malawi | 5973 (4863-7246) | 159.331 (130.635-190.829) | 13150 (10021-17056) | 179.915 (140.229-227.226) | 0.29 (0.12 to 0.47) |
| Malaysia | 4551 (3013-5813) | 51.256 (33.548-66.117) | 14185 (10511-17727) | 52.101 (38.15-64.946) | -0.16 (-0.3 to -0.01) |
| Maldives | 27 (20-36) | 34.379 (26.364-45.86) | 61 (47-80) | 19.4 (14.753-24.76) | -2.22 (-2.33 to -2.1) |
| Mali | 6918 (5667-8440) | 184.553 (151.775-222.231) | 14722 (10884-19758) | 176.805 (134.331-231.641) | -0.03 (-0.11 to 0.04) |
| Malta | 533 (490-571) | 124.487 (114.508-133.385) | 739 (644-835) | 74.121 (65.356-83.041) | -1.67 (-1.82 to -1.52) |
| Marshall Islands | 6 (5-8) | 37.284 (27.636-48.474) | 17 (12-24) | 46.507 (31.611-63.08) | 0.91 (0.77 to 1.04) |
| Mauritania | 478 (374-617) | 48.94 (38.473-63.379) | 802 (539-1163) | 38.474 (25.839-55.178) | -1.14 (-1.27 to -1) |
| Mauritius | 739 (700-778) | 103.93 (98.306-109.516) | 872 (802-926) | 47.693 (44.071-50.571) | -1.52 (-2.3 to -0.74) |
| Mexico | 13824 (13463-14137) | 33.424 (32.417-34.257) | 35647 (31180-40528) | 28.417 (24.925-32.279) | -0.42 (-0.58 to -0.26) |
| Micronesia (Federated States of) | 20 (15-27) | 40.542 (29.91-52.893) | 36 (24-50) | 45.676 (31.325-62.267) | 0.46 (0.42 to 0.49) |
| Monaco | 74 (49-102) | 104.011 (68.037-144.202) | 107 (51-241) | 105.293 (49.742-238.311) | 0.11 (0.08 to 0.14) |
| Mongolia | 600 (369-808) | 55.088 (34.497-73.939) | 687 (520-882) | 28.314 (21.534-36.611) | -2.94 (-3.4 to -2.49) |
| Montenegro | 493 (386-618) | 78.921 (61.887-98.639) | 904 (706-1163) | 91.668 (71.767-117.803) | 0.52 (0.41 to 0.62) |
| Morocco | 3511 (2559-4556) | 25.749 (18.409-33.339) | 9344 (6446-12297) | 28.461 (19.802-36.99) | 0.39 (0.24 to 0.55) |
| Mozambique | 3279 (2550-4252) | 57.415 (45.12-73.937) | 6952 (5039-9534) | 63.82 (47.234-85.52) | 0.66 (0.55 to 0.78) |
| Myanmar | 7880 (5665-10603) | 34.061 (25.379-44.545) | 11751 (8315-17877) | 24.446 (17.433-37.653) | -1.27 (-1.36 to -1.17) |
| Namibia | 207 (171-253) | 32.93 (27.047-39.956) | 484 (365-638) | 35.764 (27.692-45.912) | 0.08 (-0.16 to 0.33) |
| Nauru | 3 (2-4) | 53.845 (37.253-72.45) | 3 (2-5) | 56.221 (36.905-79.869) | 0.17 (0.04 to 0.3) |
| Nepal | 2336 (1532-3627) | 26.489 (17.37-40.699) | 5290 (3516-10650) | 23.827 (15.956-47.536) | -0.34 (-0.59 to -0.1) |
| Netherlands | 22986 (21426-24312) | 113.891 (106.019-120.316) | 27887 (25223-30326) | 75.986 (69.222-82.314) | -1.23 (-1.39 to -1.08) |
| New Zealand | 3204 (2923-3471) | 80.43 (73.566-86.79) | 4944 (4426-5438) | 56.901 (51.067-62.425) | -1.41 (-1.71 to -1.1) |
| Nicaragua | 191 (164-221) | 12.538 (10.714-14.523) | 620 (506-771) | 12.779 (10.434-15.802) | 0.26 (0.03 to 0.49) |
| Niger | 968 (716-1442) | 36.356 (26.969-53.825) | 2240 (1544-3587) | 28.576 (19.925-45.294) | -0.95 (-1.06 to -0.85) |
| Nigeria | 5784 (4471-8289) | 13.778 (10.787-19.486) | 11914 (9367-15548) | 14.163 (11.346-18.59) | 0.13 (0.08 to 0.18) |
| Niue | 1 (1-1) | 33.048 (24.795-41.29) | 1 (1-1) | 39.849 (28.499-50.868) | 0.53 (0.46 to 0.6) |
| North Macedonia | 1821 (1532-2203) | 99.285 (83.837-120.028) | 3340 (2536-4340) | 100.882 (77.582-129.76) | -0.02 (-0.28 to 0.24) |
| Northern Mariana Islands | 6 (5-10) | 31.855 (24.458-51.096) | 38 (28-43) | 71.836 (54.604-81.426) | 3.91 (3.32 to 4.5) |
| Norway | 7667 (7256-7997) | 109.517 (104.182-113.95) | 7075 (6357-7591) | 66.712 (60.598-71.441) | -1.62 (-1.77 to -1.47) |
| Oman | 247 (176-336) | 38.404 (27.473-52.615) | 541 (413-693) | 30.005 (23.38-37.876) | -0.49 (-0.71 to -0.27) |
| Pakistan | 49944 (39990-60752) | 91.685 (72.519-111.519) | 120139 (94834-157045) | 103.367 (81.046-136.354) | 0.13 (-0.04 to 0.31) |
| Palau | 2 (1-2) | 14.747 (11.03-18.585) | 3 (2-4) | 14.104 (10.275-18.486) | -0.1 (-0.17 to -0.03) |
| Palestine | 684 (493-910) | 82.171 (59.931-108.346) | 1622 (1284-2012) | 66.774 (53.469-83.707) | -0.8 (-1 to -0.6) |
| Panama | 336 (313-363) | 22.836 (21.24-24.691) | 1007 (788-1213) | 22.766 (17.835-27.457) | -0.12 (-0.31 to 0.07) |
| Papua New Guinea | 478 (267-725) | 25.841 (15.021-38.165) | 1548 (890-2387) | 29.177 (17.176-44.554) | 0.48 (0.43 to 0.52) |
| Paraguay | 466 (371-601) | 21.704 (17.335-27.729) | 1629 (1169-2149) | 28.729 (20.71-37.775) | 1.09 (1.01 to 1.17) |
| Peru | 3708 (3031-4547) | 31.652 (25.726-39.094) | 8744 (6416-11668) | 26.244 (19.23-35.037) | -0.82 (-1.05 to -0.59) |
| Philippines | 5864 (4916-7045) | 19.321 (16.197-23.402) | 17359 (14125-22495) | 20.622 (16.827-27.656) | 0.29 (0.24 to 0.33) |
| Poland | 50791 (48852-52662) | 114.682 (110.255-118.929) | 100472 (90643-110280) | 136.938 (123.783-150.316) | 0.34 (0.18 to 0.49) |
| Portugal | 13602 (12730-14479) | 98.427 (92.239-104.338) | 20689 (18671-22758) | 81.966 (74.796-90.457) | -0.55 (-0.65 to -0.44) |
| Puerto Rico | 2009 (1865-2142) | 55.981 (52.09-59.691) | 3173 (2593-3744) | 44.537 (36.516-52.846) | -0.64 (-0.82 to -0.47) |
| Qatar | 104 (75-138) | 107.864 (82.449-140.004) | 530 (363-798) | 63.49 (44.645-93.271) | -1.71 (-2.15 to -1.27) |
| Republic of Côte d'Ivoire | 1963 (1480-2424) | 52.789 (40.281-63.332) | 5223 (3863-7501) | 49.169 (37.225-68.831) | -0.36 (-0.49 to -0.24) |
| Republic of Korea | 16705 (13565-19362) | 60.492 (49.162-69.917) | 34899 (26630-42731) | 37.176 (28.392-45.454) | -1.79 (-1.88 to -1.69) |
| Republic of Moldova | 3252 (3018-3523) | 74.122 (68.926-80.417) | 4297 (3829-4810) | 70.623 (62.948-79.036) | -0.42 (-0.64 to -0.21) |
| Republic of South Sudan | 1369 (1003-1915) | 54.43 (40.339-75.603) | 1967 (1371-2670) | 53.012 (37.488-72.621) | -0.25 (-0.41 to -0.1) |
| Romania | 24609 (22797-26267) | 86.208 (80.105-91.928) | 39087 (34634-44244) | 105.519 (93.021-119.115) | 0.53 (0.41 to 0.65) |
| Russian Federation | 136386 (132203-140477) | 74.021 (71.769-76.283) | 150908 (136510-166266) | 62.389 (56.5-68.67) | -1.05 (-1.39 to -0.7) |
| Rwanda | 2060 (1589-2569) | 73.91 (57.741-91.336) | 2985 (1987-4543) | 49.689 (33.545-75.066) | -2.01 (-2.26 to -1.75) |
| Saint Kitts and Nevis | 27 (25-29) | 71.847 (66.566-76.475) | 39 (32-45) | 59.835 (49.906-68.437) | 0.09 (-0.14 to 0.32) |
| Saint Lucia | 67 (63-71) | 79.663 (75.042-84.478) | 144 (117-172) | 60.311 (49.195-71.907) | -0.9 (-1.13 to -0.67) |
| Saint Vincent and the Grenadines | 37 (34-40) | 52.068 (48.037-55.944) | 73 (64-84) | 51.885 (45.769-58.975) | 0.35 (0.12 to 0.59) |
| Samoa | 22 (17-32) | 26.934 (21.059-37.751) | 35 (25-52) | 25.202 (18.028-36.027) | -0.27 (-0.32 to -0.21) |
| San Marino | 52 (42-65) | 144.741 (114.933-180.704) | 57 (38-81) | 72.926 (46.695-106.373) | -1.22 (-1.59 to -0.86) |
| Sao Tome and Principe | 46 (38-56) | 72.059 (59.503-88.155) | 98 (71-128) | 88.607 (64.646-117.073) | 0.74 (0.64 to 0.84) |
| Saudi Arabia | 2254 (1579-3304) | 40.837 (28.571-57.743) | 6819 (4625-12353) | 35.735 (25.01-59.709) | -0.65 (-0.79 to -0.52) |
| Senegal | 1668 (1277-2092) | 52.847 (41.116-64.952) | 3484 (2587-4721) | 45.745 (34.395-61.139) | -0.67 (-0.76 to -0.58) |
| Serbia | 11873 (9473-14614) | 112.234 (90.271-137.312) | 17203 (12997-22015) | 102.12 (76.654-130.075) | -0.29 (-0.37 to -0.21) |
| Seychelles | 58 (51-67) | 102.982 (89.396-118.82) | 86 (72-107) | 76.379 (63.773-95.635) | -0.49 (-0.69 to -0.29) |
| Sierra Leone | 937 (692-1187) | 46.512 (34.716-58.211) | 1487 (1086-2056) | 39.121 (29.236-52.748) | -0.69 (-0.76 to -0.62) |
| Singapore | 939 (868-1011) | 44.775 (41.389-48.147) | 2161 (1928-2378) | 25.698 (22.99-28.277) | -1.82 (-2.03 to -1.61) |
| Slovakia | 6397 (5360-7665) | 105.944 (89.186-127.426) | 8199 (6403-10728) | 84.703 (66.725-110.092) | -0.53 (-0.6 to -0.45) |
| Slovenia | 2231 (2049-2398) | 90.02 (82.727-96.623) | 3935 (3373-4529) | 85.82 (73.015-99.134) | -0.41 (-0.6 to -0.23) |
| Solomon Islands | 48 (23-72) | 34.343 (17.667-50.963) | 148 (91-218) | 39.844 (24.282-58.066) | 0.58 (0.51 to 0.65) |
| Somalia | 1415 (1015-1968) | 61.018 (44.463-83.916) | 3323 (2333-4428) | 55.371 (39.459-73.3) | -0.31 (-0.36 to -0.27) |
| South Africa | 9923 (7944-12642) | 45.665 (36.053-59.229) | 23296 (20278-26651) | 49.848 (43.332-56.803) | 0.25 (0.05 to 0.46) |
| Spain | 81216 (75465-86366) | 146.663 (136.524-155.445) | 100340 (90134-110098) | 99.118 (90.213-108.645) | -1.21 (-1.36 to -1.06) |
| Sri Lanka | 2292 (1915-2833) | 22.474 (18.628-27.752) | 5134 (3235-7417) | 19.151 (12.233-27.583) | -0.21 (-0.41 to -0.01) |
| Sudan | 5924 (4082-9169) | 64.781 (44.226-99.89) | 10666 (7605-14485) | 55.866 (41.33-75.025) | -0.56 (-0.64 to -0.49) |
| Suriname | 87 (72-106) | 35.075 (28.812-42.831) | 212 (154-284) | 33.54 (24.454-44.824) | 0.21 (0.03 to 0.39) |
| Sweden | 12024 (11177-12801) | 77.696 (72.685-82.328) | 14047 (12300-15765) | 59.813 (52.571-67.193) | -0.43 (-0.68 to -0.18) |
| Switzerland | 5814 (5327-6324) | 55.373 (51.031-60.027) | 10887 (9555-12304) | 56.561 (49.971-63.652) | -0.08 (-0.35 to 0.19) |
| Syrian Arab Republic | 2557 (1951-3351) | 49.387 (37.652-65.41) | 6342 (4462-8796) | 49.891 (35.541-68.738) | -0.18 (-0.27 to -0.08) |
| Taiwan (Province of China) | 12652 (11781-13358) | 81.193 (75.386-85.938) | 26566 (23444-29387) | 62.785 (55.743-69.395) | -1.37 (-1.63 to -1.12) |
| Tajikistan | 598 (437-817) | 21.676 (15.58-30.466) | 954 (695-1286) | 16.37 (12.124-21.683) | -1.09 (-1.35 to -0.83) |
| Thailand | 18471 (14750-22724) | 54.279 (43.411-66.415) | 46442 (34512-61080) | 43.225 (32.126-56.589) | -1.22 (-1.37 to -1.06) |
| Timor-Leste | 60 (43-80) | 21.859 (16.136-29.416) | 175 (126-251) | 20.77 (14.924-30.508) | -0.1 (-0.31 to 0.11) |
| Togo | 596 (473-728) | 49.745 (39.927-59.795) | 1714 (1268-2322) | 44.561 (33.663-58.933) | -0.64 (-0.79 to -0.49) |
| Tokelau | 0 (0-1) | 34.214 (25.069-44.636) | 1 (0-1) | 35.003 (23.786-47.97) | 0.11 (0.05 to 0.16) |
| Tonga | 15 (10-22) | 27.002 (17.884-40.42) | 26 (16-39) | 31.922 (20.146-49.002) | 0.61 (0.47 to 0.75) |
| Trinidad and Tobago | 366 (345-385) | 44.86 (42.428-47.305) | 760 (589-965) | 38.928 (30.252-49.36) | -0.21 (-0.36 to -0.06) |
| Tunisia | 3363 (2562-4336) | 71.137 (53.811-91.137) | 8992 (6086-12695) | 69.152 (46.826-97.069) | -0.32 (-0.41 to -0.22) |
| Turkey | 36236 (27669-47341) | 106.182 (81.979-137.474) | 78534 (60067-100388) | 84.067 (64.529-106.592) | -0.9 (-1.12 to -0.67) |
| Turkmenistan | 731 (651-819) | 36.862 (32.736-41.325) | 1202 (924-1571) | 28.549 (21.993-37.009) | -1.5 (-1.84 to -1.16) |
| Tuvalu | 2 (2-3) | 34.61 (25.503-43.916) | 4 (3-5) | 39.435 (27.361-50.12) | 0.55 (0.49 to 0.61) |
| Uganda | 3818 (2887-4878) | 61.829 (47.488-78.292) | 8498 (6385-11196) | 59.951 (45.063-77.747) | -0.55 (-0.74 to -0.36) |
| Ukraine | 45613 (37695-57782) | 62.239 (51.628-78.519) | 51393 (36167-68944) | 66.199 (46.517-89.233) | -0.07 (-0.35 to 0.21) |
| United Arab Emirates | 572 (338-1007) | 120.56 (70.625-204.276) | 3065 (2250-4109) | 87.212 (67.291-110.348) | 0.09 (-0.39 to 0.57) |
| United Kingdom | 122600 (118312-125824) | 133.695 (129.22-137.068) | 110390 (101201-115963) | 80.782 (75.019-84.53) | -1.61 (-1.75 to -1.47) |
| United Republic of Tanzania | 6287 (4918-8340) | 59.397 (46.743-77.748) | 12041 (8835-16721) | 48.428 (35.725-66.071) | -0.87 (-0.94 to -0.8) |
| United States of America | 241031 (226731-252649) | 74.571 (70.424-78.136) | 407123 (371660-433093) | 68.15 (62.691-72.493) | -0.24 (-0.35 to -0.13) |
| United States Virgin Islands | 28 (22-35) | 34.077 (27.076-42.559) | 42 (31-55) | 23.214 (17.264-30.099) | -1.11 (-1.31 to -0.91) |
| Uruguay | 4759 (4427-5083) | 120.678 (112.368-128.693) | 5629 (5120-6148) | 100.178 (91.676-109.47) | -0.84 (-0.96 to -0.72) |
| Uzbekistan | 2964 (2162-3895) | 25.246 (18.048-33.605) | 7347 (5648-9467) | 27.381 (21.256-35.098) | 0.2 (-0.01 to 0.41) |
| Vanuatu | 21 (13-30) | 32.594 (21.144-46.773) | 68 (45-92) | 37.366 (25.16-50.261) | 0.43 (0.38 to 0.48) |
| Venezuela (Bolivarian Republic of) | 3586 (3408-3742) | 37.683 (35.604-39.368) | 11212 (8555-14376) | 37.852 (28.996-48.372) | -0.3 (-0.43 to -0.18) |
| Viet Nam | 8441 (6374-10808) | 21.336 (16.143-27.168) | 23807 (18185-29217) | 24.514 (18.863-30.019) | 0.46 (0.42 to 0.51) |
| Yemen | 3095 (2092-4494) | 63.966 (43.524-91.985) | 8350 (5903-11374) | 61.246 (43.307-82.599) | -0.22 (-0.27 to -0.16) |
| Zambia | 1764 (1441-2176) | 63.679 (52.154-79.034) | 5230 (3008-9788) | 74.682 (45.197-130.34) | 0.43 (0.3 to 0.55) |
| Zimbabwe | 5329 (4236-6484) | 136.234 (109.229-164.228) | 11837 (8319-15780) | 169.208 (121.98-220.278) | 0.93 (0.6 to 1.27) |

ASR, age-standardized rate; EAPC, estimated annual percentage change; UI, uncertainty interval; CI, confidence interval.
